# The Aarhus bereavement study (TABstudy)

**DOI:** 10.1007/s10654-026-01397-z

**Published:** 2026-05-30

**Authors:** Madeline Marie Marello Havn, Sören Möller, Christy Denckla, Marie Lundorff, Lea Tangelev Greve, Maja O’Connor

**Affiliations:** 1 Unit for Bereavement Research, Department of Psychology and Behavioural Sciences, Aarhus University, Bartholins Allé 11, 1351-317, Aarhus C, Denmark; 2 Epidemiology, Biostatistics and Biodemography, Department of Public Health, University of Southern Denmark, Odense, Denmark; 3 Department of Social and Behavioral Sciences, Harvard T.H. Chan School of Public Health, Boston, MA USA; 4 Aarhus University Hospital, Palle Juul-Jensens Boulevard 99, Aarhus N, DK-8200 Denmark; 5 Developmental Psychology, Department of Psychology and Behavioral Sciences, Aarhus University, Aarhus C, Denmark; 6 The Danish National Centre for Grief, Copenhagen, Denmark

**Keywords:** Cohort study, Population-based, Danish nationwide administrative registers, Bereavement

## Abstract

The Aarhus Bereavement Study (TABstudy) was established to assess the health and societal impact of bereavement and pathological grief. This is the first register-identified bereavement cohort, linked to a longitudinal survey on grief, with non-bereaved controls. The entire cohort (*N* = 68,960), followed from Danish register creation until 2030, includes annual data on socio-economic demographics, income, workforce, government financial assistance, education, incidence of somatic health conditions, incidence of psychiatric health conditions, prescription medication sales, and cause of mortality for bereaved spouses, matched controls, and all their children. The longitudinal self-reported survey (*N* = 1,227, 54% response rate) of recent spousal-loss and parental-loss in Aarhus, Denmark, from 2017 to 2018 collected data on lifestyle factors, substance use, personality, significant life changes, well-being, and psychological measures in 8 waves across 5 years. A final 10-year follow-up survey wave is planned for 2027, while register data will cover until 13 years after loss. The cohort at bereavement was on average 51 years old, 56% women, most attained secondary education or higher, and most were employed. Bereaved and control subsamples were similar on nearly all characteristics. Data are stored at Statistics Denmark. Researchers interested in collaboration should contact the Unit for Bereavement Research, Aarhus, Denmark (maja@psy.au.dk, Dr. Maja O’Connor).

**Supplementary Information:**

The online version contains supplementary material available at https://doi.org/10.1007/s10654-026-01397-z.

## Rationale

Bereavement is a major life stressor linked to elevated risks for mortality, detriments to physical health, and problems with mental health [1–3]. Such emotional stress due to grief has been linked to physical health consequences, including poorer immune function, sleep disturbances, cardiovascular disease, chronic pain, osteoarthritis, and diabetes [4–7]. Mental health problems after bereavement include depression, post-traumatic stress disorder (PTSD), and prolonged grief disorder (PGD) [8, 9].

PGD is a recently classified disorder, distinct from other post-loss mental health disorders, such as depression [10, 11]. Significant adverse reactions to grief have been studied for decades, under numerous names and varied criteria – often called prolonged grief, complicated grief, traumatic grief, or persistent complex grief [12–15]. Only recently have diagnostic manuals formally adopted diagnostic codification for PGD. The American Psychiatric Association’s Diagnostic and Statistical Manual of Mental Disorders (DSM-5-TR) included PGD in 2022, and the World Health Organization’s International Classification of Diseases (ICD-11) included the disorder in 2018 [15–17]. The prevalence of PGD is estimated to be between 3% and 13% of bereaved adults, with higher prevalence estimates for those bereaved by unnatural deaths [18–21]. This highlights the pressing need for rigorous research, methods, and measures of PGD given the recent diagnostic codification and fluctuating prevalence estimates [22–24].

PGD research has largely developed along two parallel methodological traditions. A substantial body of literature is based on survey data, identifying key risk factors through self-reported measures [25, 26]. While these studies provide valuable insight into subjective experiences, they often rely on retrospective recall for mental disorders or assess grief reactions long after the loss has occurred, increasing susceptibility to recall bias and misclassification. While the register-based bereavement literature offers access to prospectively collected data and precise temporal ordering for outcomes such as psychiatric diagnoses, healthcare utilization, and mortality. Specifically, the Nordic register infrastructure provides a unique opportunity for bereavement research, enabling linkage of population-wide data for health, social, and demographic variables with virtually complete follow-up over time. This facilitates register-based research examining mental health outcomes following bereavement, such as risk of psychiatric disorders, antidepressant use, and suicide [27–31]. Several large-scale Nordic register cohorts have examined specific loss contexts, such as suicide [32, 33], or specific relationships lost, such as parents’ loss of a pre-natal or young child [34–36], young children’s loss of a parent or close relative [37–41], and loss of a spouse [42, 43]. However, register-based cohorts typically lack self-reported measures of grief severity, psychological symptoms, and subjective reports of functional impairment. This limits the ability to capture lived bereaved experiences and clinically relevant factors occurring outside of register datasets. Despite the complementary strengths of these two methodological approaches, relatively few bereavement studies have integrated survey data with register data within the same cohort. There is a gap in the literature for studies that can simultaneously leverage register data with self-reported accounts.

The Aarhus Bereavement Study (TABstudy, ‘tab’ also meaning ‘loss’ in Danish) was established in the greater Aarhus area of Denmark in 2017, to examine bereavement and health outcomes through the combination of register data and survey data. This cohort was designed to be a register-identified population, combined with longitudinal self-reported surveys to answer a range of bereavement-related research questions. Previous register cohorts combined with bereavement surveys have been gathered for grieving young children [40, 44], or for specific types of loss such as natal-loss [45], cancer-loss [46], and wife-loss for elderly men [47]. This is the first register-based cohort with the added strengths of multiple bereavement types for adults, matched non-bereaved controls for comparison, and principally, the combination of longitudinal self-report surveys with register data. This cohort was created to explore the long-term psychological, social, and health-related associations of bereavement, including:

(i) Overall morbidity and mortality of bereaved adults, particularly cardiovascular events, immune system dysregulation, and healthcare usage – to validate findings in prior literature with a robust comparison to non-bereaved controls [1–7].
(ii) Risks and protective factors for adverse grief reactions – to validate recent findings from meta-analyses on surveys [25, 26].
(iii) The healthcare demands of the recently bereaved, to articulate a support plan for professionals to address such needs.
(iv) Development and persistence of mental health conditions within the context of bereavement, particularly PGD, depression, and PTSD [8, 9].
(v) Various types of loss, specifically spousal- and parental-loss, with the ability to evaluate entire family units as cases [48].
(vi) Explore survey and register methodologies of the same bereaved individuals, specifically agreement for health indicators between data sources, and tracking of survey non-responders. To improve the rigor of bereavement literature and improve the prevalence rates of bereavement-related conditions [24].

By linking psychological questionnaires and register-based health data, we hope this cohort will provide a holistic understanding of the consequences of grief over time.

## Cohort

### Setting

**Fig. 1:**
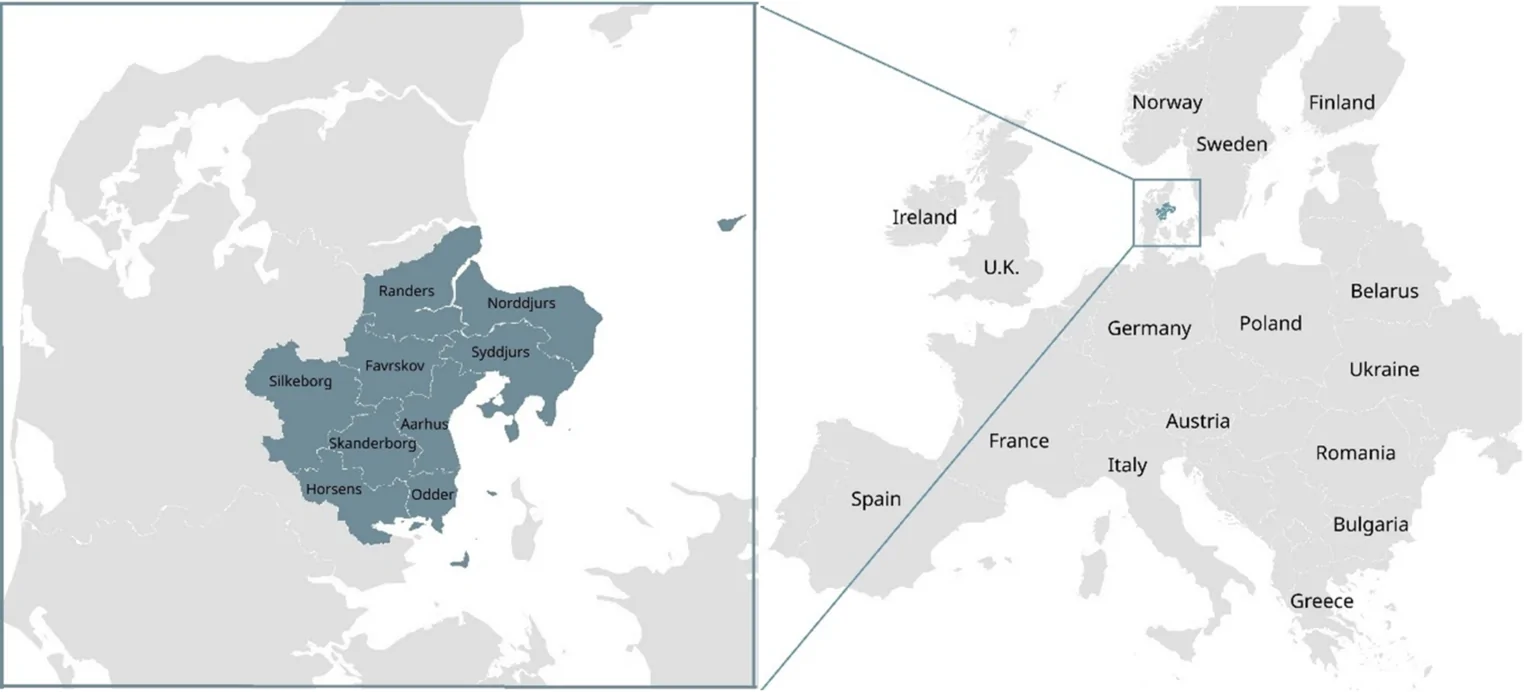
The Aarhus Bereavement Study recruitment area: The greater Aarhus area of nine municipalities from Central Denmark Region, Denmark

In 2017, the cohort was established via the Danish Civil Registration System (DCRS), identifying recently deceased individuals and linking to their surviving family members and matched controls. Like other Scandinavian settings, Denmark is a highly educated, high-income country known for its universal healthcare system and high-quality national registers [49, 50]. The 2017 life expectancy for adults in Denmark is 84.3 years for women and 80.9 years for men [51]. The study primarily draws survey participants from the greater Aarhus area, encompassing nine municipalities (see Fig. 1). The greater Aarhus area was chosen due to the mix of urban and rural municipalities still within a close location to the research lab for feasibility and participant familiarly, a important variable for survey response rates [52]. During the primary recruitment year of 2017, the population size of the greater Aarhus area was 824,836 individuals, of whom 7% are non-Danes and 17% aged 65 or older. Similarly, the entire population of Denmark in 2017 was 5,748,769 individuals, of whom 8% are non-Danes and 19% aged 65 or older [51]. Included in this area is Aarhus municipality, which is part of the Central Denmark Region and includes Denmark’s second-largest city, also named Aarhus. Aarhus city has a relatively young and well-educated population, predominantly due to Aarhus University [53]. This demographic composition of Aarhus city and municipality was the reason we targeted the greater Aarhus area so that the inclusion of nearby municipalities increased generalizability. At the time The Aarhus Bereavement Study was initiated in 2017 there were no established municipality-based grief support systems for citizens of Aarhus. This mirrored the rest of Denmark, where no national guidelines for managing bereavement and loss were available in 2017. Within Aarhus municipality now in 2026, there are some established grief social support networks, through the church and volunteer organizations, although more support is focused towards young children, additionally through counselling, and the Danish Cancer Society [54].

### Population

The TABstudy cohort consists of five subsamples:

(A) *Deceased index subsample* Recently deceased married individuals who left behind a living spouse residing in the greater Aarhus area. Cohort statistics do not include this index subsample unless specifically stated.
(B) *Spousal-bereaved subsample* The spouses of the deceased index subsample, drawn from the greater Aarhus area.
(C) *Parental-bereaved subsample* All children of the spousal-bereaved subsample and all children of the deceased index subsample.
(D) *Control-spouse subsample* Individuals who are age- and gender-matched to the spousal-bereaved subsample, drawn from the general Danish population.
(E) *Control-child subsample* All children of the control-spouse subsample.

Complete registry data is available for the entire cohort of 68,960 individuals, consisting of 7,017 bereaved individuals (representing 2,006 bereaved family units consisting of parent and adult-child(ren)) and 61,943 control individuals (representing 18,611 control family units). Additional survey data is available from 1,227 bereaved individuals from the spousal-bereaved subsample (B) and parental-bereaved subsample (C). Survey data is available from at least one person for 873 bereaved families, and available from two or more persons for 252 bereaved families.

The spousal-bereaved subsample (B) was identified through monthly extractions from DCRS between January 2017 and March 2018 [49]. Recently deceased individuals (A) were identified first through the death registry, then any living spouse who resided in the greater Aarhus area was selected for cohort inclusion. Eligibility required that the bereavement occurred within the previous five weeks, meaning some index death dates took place in late 2016. All of subsample B was invited to the survey (see Recruitment for survey).

For the deceased index subsample (A) and the spousal-bereaved subsample (B), all children – living and deceased – were identified for the parental-bereaved subsample (C). This means the parental-bereaved subsample includes bereavement types of biological-parent loss, stepparent loss, and adopted-parent loss. Only a selection of subsample C was invited to the survey (see Recruitment for survey).

For each spousal-bereaved individual (B), approximately ten age- and gender-matched controls (D) were selected from the DCRS. This control-spouse subsample was married at the index date, resided anywhere in Denmark, and used primarily as comparisons for the spousal-bereaved subsample.

For the control-spouse subsample (D), all children – living and deceased – were identified for the control-child subsample (E) and used primarily as comparisons for the parental-bereaved subsample (B).

### Recruitment for survey

Following recommended methods for increasing response rates in bereavement samples [52, 55, 56] recruitment occurred in two stages: first by a pre-notice letter and then an official invite by phone. One month after loss, all bereaved spouses (subsample B) were mailed the pre-notice letter (*n* = 2,006 total) - primarily a condolence letter, with information about the upcoming survey and stating researchers would reach out by phone. The majority of individuals had a known associated phone number. If no phone number was available, a pre-stamped contact card was also sent requesting a reliable phone number. At two months post-loss, the spousal-bereaved (subsample B) were telephoned (*n* = 1,560 invited) by trained research assistants and officially invited to participate in the study. The reasons for not being invited to the survey study were unknown phone numbers (*n* = 253), unable to be reached through known phone numbers (*n* = 190), and incorrect phone numbers (*n* = 3). Invited individuals could refuse over the phone (*n* = 478) or decline by letter (*n* = 96). Informed consent was completed over the phone and in writing before surveys were sent out to those who agreed to participate (*n* = 986 agreed), and the survey was returned through their self-selected method of a letter or online link. The first survey wave from the spousal-bereaved (subsample B) there was a subsample response rate of 85% among accepters, 54% among invited, and 42% among all DCRS identified as eligible.

To examine parental-bereavement, children of the spousal-bereaved subsample were sought out for inclusion (subsample C). Due to ethical considerations (i.e., risk of informing adopted children of the deaths of an unknown biological parent), the bereaved children were only contacted through the spousal-bereaved (subsample B) who provided contact information for their adult children and passed along the first survey invitation. Therefore, it is possible that bereaved spouses (subsample B) did not participate in the survey, but their parental-bereaved children did participate (subsample C). Thus, these parental-bereaved individuals were invited to the survey via their living parent, using a snowball recruitment method (*n* = 529 invited). Surveys were sent out to all parental-bereaved individuals who agreed to participate (*n* = 400 agreed), and survey data was returned through their chosen communication method of letter or online link. In the first survey wave of the parental-bereaved (subsample C) there was a subsample response rate of 91% among accepters, 69% among invited, and 8% among all DCRS identified as eligible. The included parental-bereaved subsample followed the same study-protocol as the spousal-bereaved subsample.

The representativeness of recruited survey participants was assessed against the total known eligible index sample (see supplemental table S4). The baseline bereaved survey responders were representative of education level, average income for the year, manner of loss (natural vs. unnatural), and prior mental health conditions recorded before the bereavement date. However, our survey responders were more likely to be female (vs. male) and single (vs. currently married or cohabitating) than compared to the eligible index sample, similar to other bereavement studies which thoroughly evaluated representativeness of survey responders [57].

### Ethics

TABstudy was pre-registered at clinicaltrials.org as an observational study [NCT03049007] and ethical approval has been obtained from Aarhus University [journal number 2022–0367531, serial number 3435]. In accordance with Danish law, studies that rely solely on questionnaire and registry data do not require approval from the Danish National Committee on Health Research Ethics. TABstudy follows the General Data Protection Regulation of the European Union [2016/679] and is conducted under the surveillance of the Danish Data Protection Agency [registration number: 2015-57-0002–62908-266].

## Follow-up

**Fig. 2:**
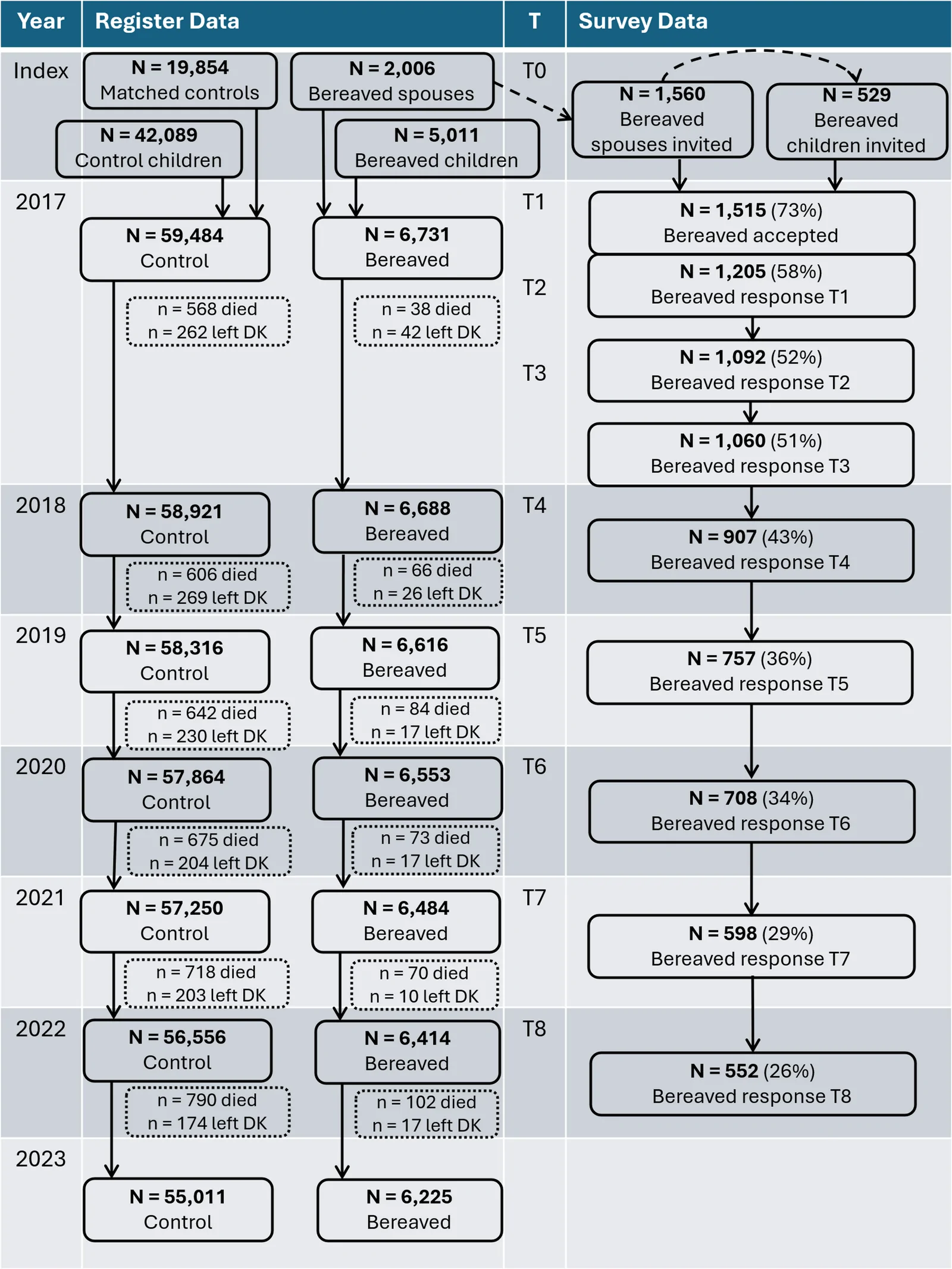
Flow Chart of The Aarhus Bereavement Study cohort (*N* = 68,960) responses and timeline of data waves. Dotted lines indicate subsample source: survey invited bereaved spouses are from the total population of bereaved spouses; survey invited bereaved children are snowball-recruited from the invited bereaved spouses. Therefore, T1 had a response rate of 80% among accepters, 58% among invited, and 17% among all DCRS identified as eligible. *N* is the number of persons, *T* is survey timepoint with additional numbers indicating each specific wave, *left DK* are persons who emigrated from Denmark that year

Registry data for each year was included for the entire cohort, coverage before the index bereavement date is available and coverage will continue until an average of 13 years after the index bereavement date, until 2030. Survey data has been collected for eight time points, across five years after the bereavement index date for both spousal- and parental-loss (see Fig. 2). From those who accepted survey participation before the first wave (*n* = 1,515), there has been a gradual decline response rates: T1, two months after (79.5%); T2, six months after (72.5%); T3, 11 months after (70.0%); T4, 18 months after (59.9%); T5, 26 months after (50.0%); T6, 38 months after (46.7%); T7, 49 months after (39.5%); and T8, 61 months after (36.4%). A final survey time point will be completed for a 10-year follow-up, circa the year 2027.

To minimize attrition over time, the TABstudy implemented several retention strategies for the survey subsample. For the first year of follow-up (T1, T2, T3), survey late-responders were contacted via phone to encourage survey completion and inquired about their reason for not participating. From time point four and onwards, the follow-up method transitioned to text message reminders, which provided a less intrusive but effective method of prompting participation [58]. To further motivate long-term involvement, the study incorporated community outreach efforts, including public research presentations held 4 years after initial recruitment. Participants were invited to these outreach events which aimed to foster transparency, share preliminary findings, and maintain participants’ interest and trust in the research process.

Retention across survey waves in the Aarhus Bereavement Study was generally high (see Fig. 2, supplemental table S2 for attrition by type of relationship lost and by family), though loss to follow-up remains an important consideration for interpreting longitudinal findings. Among all survey waves, there were 1,285 persons classified as non-responders or dropouts. Registry data indicated that of the 1,285 non-responders, 21.6% were deceased and 1.5% had emigrated, while the remaining 76.9% of non-responders were still alive and residing in Denmark. However, dropout reasons provided by 173 survey participants indicated these factors for non-participation: too upset (> 10%), lack of resources (42%), felt they moved on (> 2%), and general desire to no longer participate (46%). For the spousal-bereaved subsample across follow-up, there was a successful retainment of similar demographics, like gender; however, dropout occurred more often for people who reported subjective poor physical health. A more detailed non-response analysis of the spousal-bereaved survey subsample has been published [59].

## Measures

### Registry records

The cohort is linked to national registers using Denmark’s unique personal identification number, which allows individual-level data to be linked across all nationwide administrative registers. This cohort has register data which includes socio-demographics, economic information, pension, workforce participation, hospital admissions for both somatic and psychiatric reasons, prescription medicine sales, and mortality with causes for mortality (see Table 1). The specific Danish registers used have been described in further detail elsewhere (e.g., positive predictive value) [60–66].

Register data starts before the bereavement date and continues until 2030, which allows for assessment of pre-existing mental health diagnoses, a risk factor for PGD [25]. Index death records can identify traumatic losses (e.g., suicide), another risk factor for multiple adverse bereavement outcomes [26].

**Table 1:** Summary of Danish registers and data extracted

Category	Register Name (Danish)	Register name (English)	Data extracted (selected)	Coverage from
Sociodemographic	Befolkningen	Population	Birth date, Children in household, Date of relocation, Marital status, Marital date, Family identification, Spouse identification, Immigrant status, Country of origin, Sex, Linkage to mother, Linkage to father, Municipality	1985
	Dødsårsagsregister	Cause of Death Register	Death date, Place of death, Age at death, Manner of death	1985
	Uddannelser	Education	Date of highest education completed, Highest education completed, Current enrolled education	1981
	Historiske Vandrnger	Historical Walks	Immigration date, Emigration date, Associated country	1985
Economic	Beskæftigelsesministeriets Forløbsdatabase	The Ministry of Employment’s Database	Monthly total employment rate, Monthly industry code for employment, Monthly employer business type, Weekly government assistance	1991
	Arbejdsklassifikationsmodulet	The Work Classification Module	Main source of income, Subject of employment including self-employed, Classification of occupation, Sector for main employment, Socio-economic status Danish classification	1976
	Indkomst	Income	Sickness benefits, Maternity benefits, Dividends and investments, Student support, Unemployment support, Housing support, Child support, Work transportation deduction, Gross income, Income after tax, Net residual, Total wage income, Total personal income, Shared income, Disability support, Pension support, Taxable income	1980
	Registerbaserede Arbejdsstyrkestatistik	Register-based Labour Force Statistics	Commuting distance to work, Municipality of work, Industry of work, Sector of work, Company identification, Workplace identification, Broad salary amount, Full- or part-time work, Potential of full- or part-time work, Main occupation, Narrow salary amount, Socioeconomic status	1980
Health	Lægemiddeldatabasen	The Medicines Database	Number of packs, Anatomical Therapeutic Chemical code (ATC) for medication, Processing date, Name of medicine, Strength, Power, Volume, Redeemed refills	2011
	Landspatientregistret	The National Patient Register	Main diagnosis ICD-10^a^, secondary diagnoses, Department code, Mode of admission, Patient type, Hospital, Date of admission, Date of discharge, Diagnosis later reclaimed	1977

*ATC* Anatomical Therapeutic Chemical identification includes the following types of medications, where asterisk means subsequent codes are included: diabetes (A10*), cardiovascular (B01*, C*), osteoporosis (G03XC01, H05AA*, M05B*), prostate disease (G04C*), thyroid disorder (H03*), additive use of analgesics (M01A*, M02A*), epilepsy (N03*), Parkinson’s (N04*), psychiatric (N02*, N05*, N06*, N07*), allergies (R01A*, R06A*), asthma and COPD (R03*)

^a^Diagnoses before the year 1989 are ICD-8 codes which have been transposed to subsequent ICD-10 coding

### Survey questionnaires

The questionnaire-based measures include diagnostic measurements for various psychological symptoms, substance use habits, physical health, wellbeing, significant life events, informal bereavement social support, and personality scales (see Table 2). We used validated measures where available in Danish and adopted relevant items or back-translated full scales when needed. Translation of scales from English to Danish was completed by two people separately, with a third person confirming by back-translating from Danish to English. A detailed overview of all questionnaires is provided in the Supplementary Material (see supplementary table S5 for the detailed schedule, and supplementary table S6 for the complete questionnaire).

**Table 2:** Self-reported survey measurement schedule of follow-ups and data collection

Topic/measures	T1	T2	T3	T4	T5	T6	T7	T8
Demographics								
Age	✓							
Gender	✓							
Educational attainment	✓							
Source of income	✓							
Marital status	✓							
Length of marriage	✓							
Number of children	✓							
Loss-related factors								
Relationship lost	✓							
Cause of death	✓							
Pre-loss illness	✓							
Caregiver burden	✓							
Other losses	✓	✓	✓					
Health behaviour								
Alcohol use	✓							
Prescription medicine intake	✓							
Daily tobacco consumption	✓							
Life								
Received social help	✓	✓	✓					
Loss chapter				✓				
Major life changes					✓	✓	✓	✓
Covid-19 well-being						✓		
Covid-19 grief						✓		
Psychosocial factors								
Grief [PG-13]	✓	✓	✓	✓	✓	✓		
Grief [PG-13-R]						✓	✓	✓
Grief [ICG-R]	✓	✓	✓	✓	✓	✓		
Grief [A-PGDs]						✓	✓	✓
Grief cognitions [GCQ]							✓	✓
Grief avoidance [DAAPGD]							✓	✓
Depression [CESD-10]	✓	✓	✓	✓	✓	✓	✓	✓
Anxiety [GAD-7]	✓	✓	✓	✓	✓	✓	✓	✓
Post-traumatic stress [PCL-5]	✓	✓	✓	✓	✓	✓	✓	✓
Well-being [WHO-5]	✓	✓	✓	✓	✓	✓	✓	✓
Loneliness [T-ILS]	✓	✓	✓	✓	✓	✓	✓	✓
Social support [CSS]	✓	✓	✓	✓			✓	✓
Centrality of event [CES]	✓	✓	✓	✓	✓	✓		
Mental and physical health [SF-12]	✓			✓	✓	✓	✓	✓
Rumination [RRQ]	✓	✓	✓					
Attachment orientation [ECR]	✓					✓		
Optimism [LOT-R]	✓				✓	✓		
Neuroticism [NEO-PI-R]	✓							
Emotion regulation [FREE]	✓							
Emotion regulation [ERQ]	✓							
Shared memory [IOS]				✓				✓
Shared memory [TMS]				✓				
Shared memory [EMQ]				✓				
Existential feelings [EFS]				✓				
Bereavement hallucinations						✓		
Sleep [PSQI]						✓		
Unrealness [EUS]							✓	✓

*A-PGDs* Aarhus prolonged grief disorder survey, *CES* centrality of event scale, *CESD-10* Center for Epidemiological Studies-Depression, *DAAPGD* depressive and anxious avoidance in prolonged grief questionnaire, *ECR* experiences in close relationship-short form, *EFS* existential feelings in grief, *EMQ* everyday memory questionnaire, *ERQ* emotion regulation questionnaire, *EUS* experienced unrealness scale, *GAD-7* generalized anxiety-7, *GCQ* grief cognitions questionnaire, *ICG-R* inventory of complicated grief-revised, *IOS* scale for inclusion of others in self, *LOT-R* life orientation test-revised, *NEO-PI-R* NEO personality inventory-revised—neuroticism only, *PCL-5* posttraumatic stress disorder checklist—civilian version, *PG-13* prolonged grief-13, *PG-13-R* prolonged grief-13 revised, *PSQI* Pittsburgh sleep quality inventory, *RRQ* Rumination-reflection questionnaire (rumination only), *SF-12* short form health survey; *TMS* transactive memory system, *WHO-5* World Health Organization 5-item well-being index

## Findings

As of September 2025, 12 peer-reviewed research articles have been published on TABstudy survey data (see supplementary table S1). An updated list of all completed and on-going studies can be found on the research unit website (https://psy.au.dk/en/bereavement-research). PGD has been the most examined outcome, followed by depression and post-traumatic stress symptoms.

### Baseline

Based on risk factors identified by meta-analyses for adverse bereavement outcomes [25, 26], the following pre-loss characteristics are present in our cohort: female gender, death of partner, only primary school education, income lower than the national third decile, single marital status, unnatural death of loved one, and depression diagnosis before loss (see Table 3). Notably, only 1% of the bereavement subsample had a diagnosis of depression before their loss.

**Table 3:** Summary of pre-loss characteristics for the bereaved subsample in 2017 (*N* = 6,693)

Pre-loss characteristic	*n*	%
Single marital status	1,565	23.38
Death of partner	2,006	29.97
Violent/Unnatural death	162	2.42
Female gender	3,714	55.49
Low income	1,778	26.57
Low education	1,949	29.12
Depression diagnosis	36	0.54

Low income is defined as a disposable income at or below the national third decile for 2017. Low education is defined as only primary school completion. Depression diagnosis includes a diagnosis of ICD-10 code F30-39

**Table 4:** Characteristics of the Aarhus bereavement study cohort in 2017

	Total cohort	Bereaved	Control
	(*N* = 65,434)	(*n* = 6,676)	(*n* = 58,758)
Age Median (IQR)	52 (43–66) years	51 (43–63) years	52 (43–63) years
Average birth year	1964	1964	1964
Gender			
Male	28,844 (44%)	2962 (44%)	25,882 (44%)
Female	36,590 (56%)	3714 (56%)	32,876 (56%)
Marriage status^a^			
Single	15,917 (24%)	1565 (23%)	14,352 (24%)
Widowed	6169 (9%)	1647 (25%)	4522 (8%)
Divorced	8830 (14%)	637 (10%)	8193 (14%)
Married	34,518 (53%)	2827 (42%)	31,691 (54%)
Years of marriage			
Median (IQR)	20.6 (10.8–35.6)	16.8 (8.8–25.4)	20.8 (10.9–36.6)
Children living at home			
M (SD)	0.77 (2.31)	0.74 (1.06)	0.78 (2.41)
Ethnicity			
Danish Origin	62,857 (96%)	6448 (97%)	56,409 (96%)
Immigrant^b^	2577 (4%)	228 (3%)	2349 (4%)
Highest education			
Primary	15,450 (24%)	1623 (24%)	13,830 (24%)
Secondary	27,673 (42%)	2916 (44%)	24,757 (42%)
Tertiary	21,529 (33%)	2053 (31%)	19,476 (33%)
Employment status			
Not working	9541 (15%)	959 (14%)	8582 (15%)
Working	37,082 (57%)	3870 (58%)	33,212 (57%)
Pension and working	417 (1%)	58 (1%)	359 (1%)
Pension	19,166 (29%)	1841 (28%)	17,325 (29%)
Disposable income in DDK			
Median (IQR)	236k (159k −323k)	243k (170k – 325k)	235k (158k – 322k)
Government Assistance			
Unemployment Median (IQR)	8520 (13%) 30 (11–52) weeks	886 (13%) 30 (10–52) weeks	7634 (13%) 30 (11–52) weeks
Health-related Median (IQR)	8114 (13%) 13 (4–35) weeks	988 (15%) 13 (5–36) weeks	7126 (12%) 12 (4–35) weeks
Pension Median (IQR)	20,410 (31%) 52 (52–52) weeks	1979 (30%) 52 (52–52) weeks	18,431 (31%) 52 (52–52) weeks

N and percentages are for the current population alive and living in Denmark for 2017

^a^Marriage status data is for 2017, therefore some bereaved persons have not yet been exposed to spousal-loss which occurred in early 2018

^b^Top four countries of origin in descending order for immigrants: Turkey, Germany, Pakistan, and Bosnia-Herzegovina

The total cohort in 2017 were primarily 52 years old, therefore born in 1964, married women, who had attained secondary education, and the majority were working. When examining the bereaved subsamples (B, C) in comparison to the control subsamples (D, E) for 2017, there are mainly similarities of their characteristics (see Table 4, see supplementary table S3 for characteristics by type of relationship lost). As expected, the controls were matched on age and gender and therefore no differences were detected between bereaved. Notable differences between the bereaved and controls include that on average controls had longer marriages by 4 years and controls had slightly lower yearly income (Kr. 8,000 DKK, or $1,228 USD converted in July 2017).

## Strengths and limitations

The cohort of TABstudy offers several key strengths that enhance its value for both clinical and population-based research. First, the cohort benefits from extensive data coverage available through individual-level linkage via Denmark’s unique personal identification numbers. This register linkage allows for cohort integration with supplementary registers if desired. Participation rates in the bereavement subsample of self-report surveys are high, with a fairly representative sample. The cohort further enables differentiation between types of bereavement (e.g., spousal vs. parental) and loss characteristics (e.g., traumatic vs. non-traumatic loss), while also allowing for control comparisons between bereaved and non-bereaved individuals. The combination of self-reported longitudinal survey data and prospective registry data provides a unique framework for evaluating mental health outcomes, healthcare utilization, and risk factors across time. The linkage between survey responses and register data will provide opportunities for cross-validation of exposures and outcomes [67], the ability to proxy missing data [68], and increase methodological rigor, which is needed in bereavement literature [24, 69].

Despite its strengths, the cohort also presents limitations. The bereavement subsample is drawn primarily from the greater Aarhus area, which may limit the generalizability of findings to other geographic regions within Denmark or internationally, especially given the influence of the local university, increasing the municipality’s average education level. The parentally bereaved subsample contains only individuals who at index have lost only one parent, as snowball recruitment was completed through the remaining living parent; this may limit generalizability to individuals who have lost both or multiple parents. Additionally, due to ethical restrictions, survey responses for parentally bereaved individuals may have selection bias towards individuals who have a closer relationship with their living parent due to the nature of snowball recruitment. Our cohort is limited primarily to adults’ loss of a spouse or a parent, which limits generalizability to other relationships lost, like the contexts of child-loss, sibling-loss, or young child loss of a parent.

Secondly, access to registry data is restricted by complex approval procedures that require affiliation with a Danish research institution, posing a barrier to broader collaboration. There are also risks of bias when conducting Danish register-based research, including selection bias, information bias, and the common off-label use of psychiatric prescriptions [70–74].

Third, because the Danish healthcare system utilizes the previous ICD-10 diagnostic model, this cohort is restricted to only subjective, self-reported questionnaires on PGD. Self-reported questionnaires are subject to response bias, non-response bias, recall bias, social desirability bias, and potentially inaccurate classification of psychiatric conditions [24, 75–77]. Therefore, presently, there is no available data on PGD from registered diagnoses or structured interview assessments for this cohort. Future work will address this potential concern with a detailed evaluation of discrepancies between survey and register data for mental health [For example see, 78]. The detailed observational data of TABstudy can still serve as a valuable reference point or comparison to other PGD studies, particularly those involving interventions or treatments of bereaved persons.

Finally, while the repeated survey waves enhance the study’s ability to capture changes over time, the length and frequency of these follow-up points may have increased participant burden, potentially contributing to survey attrition during the survey coverage period. Future work will explicitly dive into this attrition concern for the entire cohort, utilizing both survey and register data. For now, attrition bias has been estimated with only survey data on the spousal-bereaved subsample of this cohort, indicating survey attrition occurs when a survey participant’s physical health is poor [59]. Survey attrition due to poor physical health is a common finding across cultures [79–82], and was a motivating factor to utilize both survey data and register data for this cohort.

## Data access

Our cohort is open to collaboration, please contact the Unit for Bereavement Research, Aarhus, Denmark (maja@psy.au.dk, Dr. Maja O’Connor). Researchers interested in using the cohort data must submit a formal study proposal, which requires approval from the principal investigator, Statistics Denmark (as the registry data controller), and the data processing office at the Centre for Integrated Register-based Research at Aarhus University (CIRRAU). Due to the sensitivity of both survey and registry data, there are two levels of access. First, registry data access is limited to approved Danish research institutions and individual researchers must then be approved for specific projects. Second, access to survey data requires entering a research collaboration with Aarhus University. All data access follows a strict “need-to-know” principle, researchers may only access the data necessary for answering their approved research questions.

## Supplementary Information

Below is the link to the electronic supplementary material.

Supplementary Material 1
