# Supplementary material for: The Aarhus bereavement study (TABstudy)
Source: Eur J Epidemiol. 2026 May 30;41(8):1029–41. doi: 10.1007/s10654-026-01397-z (PMC13612703; doi:10.1007/s10654-026-01397-z)
Supplement: Supplementary file 1 [file 10654_2026_1397_MOESM1_ESM.docx]

Supplemental Materials

For- Cohort Profile: The Aarhus Bereavement Study (TABstudy)

By- Madeline Marie Marello, Sören Möller, Christy Denckla, Marie Lundorff, Lea Tangelev Greve, and Maja O’Connor

At- European Journal of Epidemiology

**Table of Contents**

[1	The Aarhus Bereavement Study (TABstudy) overview of previous findings	2](#_Toc222831985)

[1.1	Table S1 – Findings of previous TABstudy papers	2](#_Toc222831986)

[1.2	Bibliography of TABstudy peer reviewed research articles	3](#_Toc222831987)

[2	Supplementary Cohort Results	6](#_Toc222831988)

[2.1	Table S2 – Survey attrition, detailed by relationship lost and family groupings	6](#_Toc222831989)

[2.2	Table S3 – Characteristics of cohort in 2017 split by relationship lost	7](#_Toc222831990)

[2.3	Table S4 – Representativeness of survey participants	8](#_Toc222831991)

[3	Overview of questionnaires and items for the TABstudy	9](#_Toc222831992)

[3.1	Table S5 – Timing of questionnaires across 8 waves	9](#_Toc222831993)

[3.2	Table S6 – Summary of core battery and other questionnaires	10](#_Toc222831994)

[3.3	Full length questionnaires	12](#_Toc222831995)

# 1 The Aarhus Bereavement Study (TABstudy) overview of previous findings

## 1.1 Table S1 – Findings of previous TABstudy papers

Listed in alphabetical order. These published papers only include the survey subsample data of the cohort.

| **Authors (Year)** | **Sample size** | **Relationship lost** | **Time points** | **Scales** | **Conclusions** |
| --- | --- | --- | --- | --- | --- |
| Brookman, R., et al. (2024) | 552 | Mixed | T1 – T4 | PG-13  CES | Higher scores of event-centrality and grief symptoms for spousal-bereaved than parental-bereaved. Event-centrality predicts grief symptoms. |
| Harris, C. B., et al. (2023) | 892 | Mixed | T1, T4 | PG-13  CES  ECR-SF  IOS  TMSS-C  EMQ | More intense symptoms of grief for spousal-bereaved than parental-bereaved. Predictors of high grief scores were merged identity with deceased, younger age, and memory difficulties. |
| Johannsen, M., et al. (2022)* | 82 | Mixed | T3, T4 | PG-13  CESD-10  PCL-5  GAD-7  WHO-5 | 8 weeks of Compassion-Focused Therapy is not an efficacious treatment for prolonged grief symptoms. |
| Komischke-Konnerup, K. B., et al. (2023) | 1224 | Mixed | T1 – T5 | PG-13  CESD-10  PCL-5 | High grief scores predict later high scores of PTSD and depression, but not visa versa. Higher grief, depression, and PTSD was associated with spousal-bereavement and younger age. |
| Lenferink, L. I. M., et al. (2023) | 257 dyads | Mixed, Family units | T1 – T4 | PG-13 | Parent grief predicts child grief but not visa versa. |
| Lundorff, M. et al. (2020) | 857 | Spousal-bereaved | T1 – T3 | PG-13  ICG-R  PCL-5  LOT-R  NEO  SF-12 | Four grief trajectories emerged: *resilient* of stable low symptoms, *moderate-stable* of stable moderate symptoms, *recovery* of high symptoms decreasing, and *prolonged grief* of stable high symptoms. Similar proportions of gender across groups. |
| Lundorff, M., et al. (2021) | 753 | Spousal-bereaved | T1 – T3 | PG-13  ICG-R  PCL-5 | Higher prolonged grief disorder prevalence estimated when using single time point than dual-point, and at time criterion the dual-point prevalence did not differ. |
| Maccallum, F., et al. (2021) | 741 | Spousal-bereaved | T1, T3 | PG-13  ICG-R | Many similarities in prolonged grief symptoms for men and women, with limited variation in grief outcomes between the genders. |
| O’Connor, M., et al. (2023) | 349 | Mixed | T1, T6 | A-PGDs | Separation distress and emotional distress emerged as the best fitting two factor model for both ICD-11 and DSM-5-TR criteria for prolonged grief disorder. Associated symptoms predicted functional impairment. A-PGDs is a valid scale for both diagnostic manuals. |
| Redican, E., et al. (2023) | 584 | Mixed | T1, T7 | A-PGDs  CESD-10  PCL-5  GAD-7 | There is low endorsement of the cultural deviation criterion for PGD for this Danish sample. Multiple clinically relevant PGD cases are excluded with the culture deviation requirement. |
| Thomsen, D. K., et al. (2024) | 507 | Mixed | T1 – T4 | PG-13  CESD-10  PCL-5  GAD-7  NEO  ECR-SF | Higher grief symptoms predict later less adaptive loss narratives. |
| Vang, M. L., et al. (2022) | 1093 | Mixed | T2 | PG-13  CESD-10  PCL-5  GAD-7  WHO-5 | A two-factor model of PGD was valid, matching the ICD-11 proposal of separation distress and emotional distress model. |

* This is an embedded two arm randomized control trial which recruited from TABstudy survey participants who scored over 25 on the PG-13 scale at timepoint three of the survey study, survey data was included as outcome variables for the embedded trial group.

Abbreviations: A-PGDs, Aarhus Prolonged Grief Disorder Survey; CES, Centrality of Event Scale; CESD-10, Center for Epidemiological Studies-Depression; DSM-5-TR, Diagnostic and Statistical Manual of Mental Disorders; ECR-SF, Experiences in Close Relationship-short form; EMQ, Everyday Memory Questionnaire; ERQ, Emotion Regulation Questionnaire; GAD-7, Generalized Anxiety-7; ICD-11, International Classification of Disease; ICG-R, Inventory of Complicated Grief-Revised; LOT-R, Life Orientation Test-Revised; NEO, NEO Five-Factor Inventory (neuroticism only); PCL-5, Posttraumatic Stress Disorder Checklist – Civilian Version; PG-13, Prolonged Grief-13; RRQ, Rumination-Reflection Questionnaire (rumination only); SF-12, Short Form Health Survey; WHO-5, World Health Organization 5-item Well-Being Index.

## 1.2 Bibliography of TABstudy peer reviewed research articles

Listed in Chronological order.

**2020**

Lundorff M, Bonanno GA, Johannsen M, O'Connor M. Are there gender differences in prolonged grief trajectories? A registry-sampled cohort study. Journal of Psychiatric Research. 2020; 129:168-75. <https://doi.org/10.1016/j.jpsychires.2020.06.030>

**2021**

Harris CB, Brookman R, O’Connor M. It’s not who you lose, it’s who you are: Identity and symptom trajectory in prolonged grief. Current Psychology. 2021; 42(13):11223-33. <https://doi.org/10.1007/s12144-021-02343-w>

Lundorff M, Johannsen M, O'Connor M. Time elapsed since loss or grief persistency? Prevalence and predictors of ICD-11 prolonged grief disorder using different applications of the duration criterion. Journal of Affective Disorders. 2021; 279:89-97. <https://doi.org/10.1016/j.jad.2020.09.116>

**2022**

Johannsen M, Schlander C, Farver-Vestergaard I, Lundorff M, Wellnitz KB, Komischke-Konnerup KB, O'Connor M. Group-based compassion-focused therapy for prolonged grief symptoms in adults – Results from a randomized controlled trial. Psychiatry Research. 2022; 314:114683. <https://doi.org/10.1016/j.psychres.2022.114683>

Vang ML, Prigerson HG, Elklit A, Komischke-Konnerup KB, O'Connor M. Do we all grieve the same? A multigroup test of the dimensional structure of prolonged grief disorder among Danish bereaved partners and children. Psychiatry Research. 2022; 318:114937. <https://doi.org/10.1016/j.psychres.2022.114937>

**2023**

Komischke-Konnerup KB, Vang ML, Lundorff M, Elklit A, O'Connor M. Do early symptoms of prolonged grief disorder lead to symptoms of posttraumatic stress disorder and depression? A longitudinal register-based study of the two first years of bereavement. Journal of Psychopathology and Clinical Science. 2023; 132(8): 996-1006. <https://doi.org/10.1037/abn0000859>

Lenferink LIM, O'Connor M. Grief is a family affair: examining longitudinal associations between prolonged grief in parents and their adult children using four-wave cross-lagged panel models. Psychological Medicine. 2023; 53(15):7428-34. <https://doi.org/10.1017/S0033291723001101>

Maccallum F, Lundorff M, Johannsen M, Farver-Vestergaard I, O'Connor M. An exploration of gender and prolonged grief symptoms using network analysis. Psychological Medicine. 2023; 53(5):1770-7. <https://doi.org/10.1017/S0033291721003391>

O'Connor M, Vang ML, Shevlin M, Elklit A, Komischke-Konnerup KB, Lundorff M, et al. Development and validation of the Aarhus PGD scale for operationalizing ICD-11 and DSM-5-TR Prolonged Grief Disorder. Journal of Affective Disorders. 2023; 342:201-9. <https://doi.org/10.1016/j.jad.2023.09.022>

**2024**

Brookman R, Harris CB, O’Connor M. A role for event centrality in prolonged grief disorder. Scientific Reports. 2024; 14(1):22093. <https://doi.org/10.1038/s41598-024-72754-9>

Redican E, Vang ML, Komischke-Konnerup K, Elklit A, Shevlin M, O’Connor M. Operationalization, implications and correlates of the cultural deviance criterion for ICD-11 and DSM-5 prolonged grief disorder. Death Studies. 2024; 48(9):988-99. <https://doi.org/10.1080/07481187.2023.2297061>

**2025**

Thomsen DK, Bluck S, Lundorff M, O’Connor M. “I Changed After the Death”: Symptoms of Psychopathology Predict Lower Agency and Communion Themes in Loss Narratives Over 16 Months. Cognitive Therapy and Research. 2025; 49(1):112-25. <https://doi.org/10.1007/s10608-024-10541-6>

# 2 Supplementary Cohort Results

## 2.1 Table S2 – Survey attrition, detailed by relationship lost and family groupings

*Note*. Dashed lined indicates that bereaved children were snowball sampled from bereaved spouses; it is possible that bereaved spouses did not participate but their bereaved child(ren) did. Unique bereaved families are represented by at least one person; Multi-member families are 2 or more family members; it is possible that the multi-member families include multiple bereaved children or a mix of bereaved spouse and bereaved child(ren).

## 2.2 Table S3 – Characteristics of cohort in 2017 split by relationship lost

|  | Total Cohort | Spousal Bereaved | Control Spouse | Parental Bereaved | Control Child |
| --- | --- | --- | --- | --- | --- |
|  | (N = 65,434) | (n = 1,974) | (n = 19,325) | (n = 4,702) | (n = 39,433) |
| Age  Median (IQR) | 52 (43 – 66) years | 73 (66 – 79) years | 73 (66 – 79) years | 47 (40 – 53) years | 46 (38 – 52) years |
| Average birth year | 1964 | 1946 | 1946 | 1972 | 1973 |
| Gender |  |  |  |  |  |
| Male | 28844 (44%) | 588 (30%) | 5740 (30%) | 2374 (50%) | 20142 (51%) |
| Female | 36590 (56%) | 1386 (70%) | 13585 (70%) | 2328 (50%) | 19291 (49%) |
| Marriage status* |  |  |  |  |  |
| Single | 15917 (24%) | - | 382 (2%) | 1565 (33%) | 13970 (35%) |
| Widowed | 6169 (9%) | 1608 (81%) | 4209 (22%) | 39 (1%) | 313 (1%) |
| Divorced | 8830 (14%) | - | 2902 (15%) | 637 (14%) | 5291 (13%) |
| Married | 34518 (53%) | 366 (19%) | 11832 (61%) | 2461 (52%) | 19859 (50%) |
| Years of marriage  Median (IQR) | 20.6 (10.8 – 35.6) | 46.6 (28.7 – 54.5) | 44.6 (28.3 – 52.2) | 15.0 (7.9 – 22.2) | 14.7 (8.0-22.1) |
| Children living at home  M (SD) | 0.77 (2.31) | 0.10 (0.42) | 0.12 (0.48) | 1.01 (1.13) | 1.10 (2.87) |
| Ethnicity |  |  |  |  |  |
| Danish Origin | 62857 (96%) | 1882 (95%) | 18232 (94%) | 4566 (97%) | 38177 (97%) |
| Immigrant | 2577 (4%) | 92 (5%) | 1093 (6%) | 136 (3%) | 1256 (3%) |
| Highest education |  |  |  |  |  |
| Primary | 15450 (24%) | 754 (38%) | 7017 (36%) | 869 (18%) | 6813 (17%) |
| Secondary | 27673 (42%) | 814 (41%) | 7905 (41%) | 2102 (45%) | 16852 (43%) |
| Tertiary | 21529 (33%) | 408 (21%) | 4652 (24%) | 1645 (35%) | 14824 (38%) |
| Employment status |  |  |  |  |  |
| Not working | 9541 (15%) | 87 (4%) | 772 (4%) | 872 (19%) | 7810 (20%) |
| Working | 37082 (57%) | 321 (16%) | 3378 (17%) | 3549 (75%) | 29834 (76%) |
| Pension and working | 417 (1%) | 49 (3%) | 314 (2%) | 9 (<1%) | 45 (<1%) |
| Pension | 19166 (29%) | 1549 (78%) | 15389 (80%) | 292 (6%) | 1936 (5%) |
| Disposable income in DDK  Median (IRQ) | 236k (159k – 323k) | 185k (144k – 256k) | 176k (132k –245k) | 266k (195k – 347k) | 265k (188k – 350k) |
| Government Assistance |  |  |  |  |  |
| Unemployment  Median (IQR) | 8520 (13%)  30 (11 – 52) weeks | 55 (3%)  22 (7 – 44) weeks | 496 (3%)  26 (9 – 51) weeks | 831 (18%)  30 (11 – 52) weeks | 7138 (18%)  30 (11 – 52) weeks |
| Health-related  Median (IQR) | 8114 (13%)  13 (4 – 35) weeks | 121 (6%)  13 (7 – 27) weeks | 657 (3%)  17 (5 – 51) weeks | 867 (18%)  13 (4 – 37) weeks | 6469 (16%)  12 (4 – 34) weeks |
| Pension  Median (IQR) | 20410 (31%)  52 (52 – 52) weeks | 1671 (85%)  52 (52 – 52) weeks | 16388 (85%)  52 (52 – 52) weeks | 308 (7%)  52 (52 – 52) weeks | 2043 (5%)  52 (52 – 52) weeks |
| *Note*. N and percentages are for the current population alive and living in Denmark for 2017.  * Marriage status is for 2017; therefore, some bereaved spouses have not yet been exposed to spousal-loss which occurred in early 2018. | | | | | |

## 2.3 Table S4 – Representativeness of TABsurvey: Prevalence rate of characteristics for each type of responder.

|  | **Source** | | Uncontacted | | | | | Refuser | | | | | Nonresponder | | | | | Responder at baseline | | | | |
| --- | --- | --- | --- | --- | --- | --- | --- | --- | --- | --- | --- | --- | --- | --- | --- | --- | --- | --- | --- | --- | --- | --- |
|  | n | % | n | % | Prevelance Ratio | | | n | % | Prevelance Ratio | | | n | % | Prevelance Ratio | | | n | % | Prevelance Ratio | | |
|  |  |  |  |  | Est. | CI_equ_ | CI_boot_ |  |  | Est. | CI_equ_ | CI_boot_ |  |  | Est. | CI_equ_ | CI_boot_ |  |  | Est. | CI_equ_ | CI_boot_ |
| All | 6552 | 100 | 4533 |  |  |  |  | 559 |  |  |  |  | 258 |  |  |  |  | 1202 |  |  |  |  |
| Gender | | |  |  |  |  |  |  |  |  |  |  |  |  |  |  |  |  |  |  |  |  |
| Male | 2904 | 44.3 | 2236 | 34.1 | 1.11 | 1.10-1.13 | 1.09-1.13 | 153 | 2.34 | 0.62 | 0.54-0.70 | 0.53-0.70 | 105 | 1.60 | 0.92 | 0.79-1.06 | 0.78-1.06 | 410 | 34.1 | 0.77 | 0.71-0.83 | 0.72-0.83 |
| Female | 3648 | 55.7 | 2297 | 35.1 | 0.91 | 0.89-0.93 | 0.90-0.92 | 406 | 6.20 | 1.30 | 1.25-1.37 | 1.24-1.37 | 153 | 2.34 | 1.07 | 0.96-1.18 | 0.95-1.17 | 792 | 35.1 | 1.18 | 1.14-1.22 | 1.14-1.23 |
| Marriage Status | | |  |  |  |  |  |  |  |  |  |  |  |  |  |  |  |  |  |  |  |  |
| Together* | 3202 | 48.9 | 2847 | 43.5 | 1.18 | NaN | 1.27-1.30 | 0 | 0.00 | - | - | - | 80 | 1.22 | 0.58 | 0.48-0.70 | 0.52-0.75 | 275 | 43.5 | 0.43 | 0.39-0.47 | 0.42-0.52 |
| Single | 3350 | 51.1 | 1686 | 25.7 | 0.80 | 0.78-0.82 | 0.71-0.75 | 559 | 8.53 | - | - | - | 178 | 2.72 | 1.48 | 1.37-1.60 | 1.24-1.46 | 927 | 25.7 | 1.66 | 1.63-1.68 | 1.46-1.56 |
| Education | | |  |  |  |  |  |  |  |  |  |  |  |  |  |  |  |  |  |  |  |  |
| Primary only | 1553 | 23.9 | 923 | 14.2 | 0.86 | 0.83-0.89 | 0.83-0.89 | 277 | 4.27 | 2.10 | 1.95-2.25 | 1.92-2.26 | 73 | 1.13 | 1.19 | 0.99-1.44 | 0.98-1.44 | 280 | 14.2 | 0.98 | 0.89-1.08 | 0.89-1.08 |
| Beyond | 4935 | 76.1 | 3564 | 54.9 | 1.04 | 1.04-1.05 | 1.03-1.05 | 275 | 4.24 | 0.65 | 0.60-0.71 | 0.60-0.70 | 183 | 2.82 | 0.94 | 0.87-1.01 | 0.87-1.01 | 913 | 54.9 | 1.01 | 0.98-1.04 | 0.98-1.04 |
| Income | | |  |  |  |  |  |  |  |  |  |  |  |  |  |  |  |  |  |  |  |  |
| Low | 1539 | 23.5 | 894 | 13.6 | 0.84 | 0.81-0.87 | 0.81-0.87 | 267 | 4.08 | 2.03 | 1.89-2.19 | 1.88-2.19 | 77 | 1.18 | 1.27 | 1.06-1.52 | 1.05-1.50 | 301 | 13.6 | 1.07 | 0.98-1.16 | 0.97-1.16 |
| Above | 5013 | 76.5 | 3639 | 55.5 | 1.05 | 1.04-1.05 | 1.04-1.06 | 292 | 4.46 | 0.68 | 0.63-0.74 | 0.63-0.74 | 181 | 2.76 | 0.92 | 0.85-0.99 | 0.84-0.99 | 901 | 55.5 | 0.98 | 0.95-1.01 | 0.95-1.01 |
| Manner of loss | | |  |  |  |  |  |  |  |  |  |  |  |  |  |  |  |  |  |  |  |  |
| Unnatural | 139 | 2.1 | 92 | 1.4 | 0.96 | 0.85-1.08 | 0.84-1.07 | 14 | 0.21 | 1.18 | 0.72-1.92 | 0.61-1.81 | 5 | 0.08 | 0.91 | 0.39-2.13 | 0.19-1.78 | 28 | 1.4 | 1.10 | 0.79-1.53 | 0.76-1.47 |
| Natural | 6379 | 97.9 | 4416 | 67.8 | 1.00 | 1.00-1.00 | 1.00-1.00 | 544 | 8.35 | 1.00 | 0.98-1.01 | 0.99-1.01 | 253 | 3.88 | 1.00 | 0.99-1.02 | 0.99-1.02 | 1166 | 67.8 | 1.00 | 0.99-1.01 | 0.99-1.00 |
| Prior mental health | | |  |  |  |  |  |  |  |  |  |  |  |  |  |  |  |  |  |  |  |  |
| Yes | 2015 | 30.8 | 1310 | 20.0 | 0.94 | 0.91-0.97 | 0.91-0.96 | 230 | 3.51 | 1.34 | 1.22-1.47 | 1.21-1.46 | 98 | 1.50 | 1.24 | 1.06-1.44 | 1.04-1.42 | 377 | 20.0 | 1.02 | 0.95-1.10 | 0.95-1.09 |
| No | 4537 | 69.2 | 3223 | 49.2 | 1.03 | 1.02-1.04 | 1.02-1.04 | 329 | 5.02 | 0.85 | 0.79-0.91 | 0.79-0.91 | 160 | 2.44 | 0.90 | 0.82-0.98 | 0.81-0.98 | 825 | 49.2 | 0.99 | 0.96-1.03 | 0.96-1.02 |
| *Note*. All comparisons are made against the source population (bereaved individuals alive before bereavement date). All bereaved are included together, both spousal-loss and parental-loss types.  *Abbreviations*: CI equ = confidence interval calculated with equation by Nohr & Liew (2018), CI boot = confidence interval calculated by bootstrap in R package boot.  *Colors*: Provides a quick visual overview of the table - green mean close to ideal representativeness, white means neutral, while light and dark orange indicate a large difference in representativeness (when compared to the source sample). | | | | | | | | | | | | | | | | | | | | | | |

# 3 Overview of questionnaires and items for the TABstudy

This section provides a systematic overview of the questionnaires used in The Aarhus Bereavement Study (TABstudy). The purpose is to promote transparency, replication, and the possibility of sharing with research partners. The overview covers all assessment points from T1 to T8 and includes information about time points and all the questionnaires used.

## 3.1 Table S5 – Timing of questionnaires across 8 waves

| **Timewave / Months post-loss** | **Questionnaires** |
| --- | --- |
| T1 / 2 months | - **Background information** - **Single items** - **Core battery:** PG-13, CSS, ICG-R, SF-12, GAD-7, WHO-5, CESD-10, T-ILS, PCL-5, CES - **Others**: LOT-R, RRQ, NEO-PI-R (neuroticism), ECR, FREE, ERQ |
| T2 / 6 months | - **Single items** - **Core battery:** PG-13, CSS, ICG-R, GAD-7, WHO-5, CESD-10, T-ILS, PCL-5, CES - **Others:** RRQ |
| T3/ 11 months | - **Single items** - **Core battery**: PG-13, CSS, ICG-R, GAD-7, WHO-5, CESD-10, T-ILS, PCL-5, CES - **Others:** RRQ |
| T4 / 18 months | - **Core battery:** PG-13, CSS, ICG-R, GAD-7, WHO-5, CESD-10, T-ILS, PCL-5, CES, SF-12 - **Others:** Loss chapter, TMS, EMQ, IOS |
| T5 / 26 months | - **Core battery:** PG-13, ICG-R, GAD-7, WHO-5, CESD-10, T-ILS, PCL-5, CES, SF-12. - **Others:** LOT-R, Life Changes, EFS, Press inquiry |
| T6 / 38 months | - **Core battery**: PG-13, ICG-R, GAD-7, WHO-5, CESD-10, T-ILS, PCL-5, CES, SF-12, A-PGDs. - **Others**: Life Changes, Bereavement hallucinations |
| T6b / 38 months | - **Core battery**: PG-13 R, A-PGDs, GAD-7, CESD-10, PCL-5, T-ILS, WHO-5, SF-12 - **Others**: Life Changes, Covid-19 Well-being, Covid-19 grief, LOT-R, PSQI, Bereavement hallucinations, ECR |
| T7 / 49 months | - **Core battery:** A-PGDs, GAD-7, WHO-5, CESD-10, T-ILS, PCL-5, CSS, SF-12, PG-13-R - **Others:** Life Changes, EUS, GCQ, DAAPGD |
| T8 / 61 months | - **Core battery:** A-PGDs, PG-13-R, GAD-7, WHO-5, CESD-10, T-ILS, PCL-5, CSS, SF-12. - **Others:** Life Changes, GCQ, EUS, DAAPGD, IOS |
| Abbreviations: A-PGDs, Aarhus Prolonged Grief Disorder Survey; CES, Centrality of Event Scale; CESD-10, Center for Epidemiological Studies-Depression; DAAPGD, Depressive and Anxious Avoidance in Prolonged Grief Questionnaire; DSM-5-TR, Diagnostic and Statistical Manual of Mental Disorders; ECR, Experiences in Close Relationship-short form; EFS, Existential Feelings in Grief; EMQ, Everyday Memory Questionnaire; ERQ, Emotion Regulation Questionnaire; EUS, Experienced Unrealness Scale; GAD-7, Generalized Anxiety-7; GCQ, Grief Cognitions Questionnaire; ICD-11, International Classification of Disease; ICG-R, Inventory of Complicated Grief-Revised; IOS, Scale for Inclusion of Others in Self; LOT-R, Life Orientation Test-Revised; NEO-PI-R, NEO Personality Inventory-Revised – neuroticism only; PCL-5, Posttraumatic Stress Disorder Checklist – Civilian Version; PG-13, Prolonged Grief-13; PG-13-R, Prolonged Grief-13 revised; PSQI, Pittsburgh Sleep Quality Inventory; RRQ, Rumination-Reflection Questionnaire (rumination only); SF-12, Short Form Health Survey; TMS, Transactive Memory System; WHO-5, World Health Organization 5-item Well-Being Index. | |

## 3.2 Table S6 – Summary of core battery and other questionnaires

| **CORE BATTERY** | | |
| --- | --- | --- |
| **Theme** | **Questionnaire** | **No. items** |
| Prolonged grief  (stops after T6 – new versions from T6b) | - Prolonged Grief Disorder (PG-13; Prigerson et al., 2009) - Inventory of Complicated Grief Revised (ICG-R; Prigerson & Jacobs, 2001) – NB: Selected items included to cover all diagnostic criteria formulated in ICD-11. | 13  8 |
| Prolonged grief (from T6b) | - Aarhus Prolonged Grief Disorder Survey (A-PGDs; O’Connor et al. 2025) - PG-13 revised (Prigerson et al. 2021) | 26  11 |
| Depression | - Center for Epidemiologic Studies Short Depression Scale (CES‐D 10; Radloff, 1977) | 10 |
| Anxiety | - Generalized Anxiety Disorder-7 (GAD-7; Spitzer et al., 2006) | 7 |
| PTSD | - PTSD Check List – Civilian version (PCL-5; Ruggiero et al., 2003) | 20 |
| Well-being | - WHO-5 (Heun et al., 2001) | 5 |
| Loneliness | - Three-Item Loneliness Scale (T-ILS; Hughes, Waite, Hawkley, & Cacioppo, 2004) | 3 |
| Crisis Support Scale | - Social support (CSS; Joseph et al., 1992) | 7 |
| Centrality of event | - Centrality of Event Scale (CES; Berntsen & Rubin, 2006) | 7 |
| Quality of life | - 12-Item Short Form Health Survey (SF-12; Ware et al., 1996) | 12 |

| **OTHER QUESTIONNAIRES** | | |
| --- | --- | --- |
| **Theme** | **Questionnaire** | **No. items** |
| Life changes | - Major life changes | 1 (+14) |
| Demographic information | - Education, income, housing, circumstances of loss, time married, illness up to death, length of illness, previous mental illness/co-morbidity, treatment, treatment success etc. | 20 (14) |
| Single items | - Help, alcohol, medication, and smoking consumption, earlier therapy/treatment other losses - Evaluative question - Informed consent - Relation to lost person, support, other losses - Information on RCT | 11  1  1  2(3)  1 |
| Rumination | - Rumination-Reflection Questionnaire (RRQ; Trapnell & Campbell, 1999) | 12 |
| Attachment (partner) | - Experiences in Close Relationship Scale – short (ECR; Wei et al., 2007) | 12 |
| Optimism | - Life Orientation Test - Revised (LOT-R; Scheier, Carver, & Bridges, 1994) | 10 |
| Neuroticism | - NEO Personality Inventory-Revised – neuroticism only (NEO-PI-R; Costa & McRae, 2004) | 12 |
| Emotion regulation | 1. Flexible Regulation of Emotional Expression (FREE; Burton & Bonanno, 2015) 2. Emotion Regulation Questionnaire (ERQ; Gross & John, 2003) | 8  10 |
| Shared memory | - Transactive Memory System (TMS) - Everyday Memory Questionnaire (EMQ) - Scale for Inclusion of Others in Self (IOS) | 15  13  2 |
| Life story | - Loss Chapter (Thomsen, 2018) | 7 |
| Existential feelings | - Existential Feelings in Grief (EFS; Køster & O’Connor, 2018) - *Note*: Original Danish Questionnaire, no established English translation currently | 10 |
| Press inquiries | - Are we allowed to contact you in relation to potential press inquiries? | 1 |
| Bereavement hallucinations | - Three single items on the experience of bereavement hallucinations | 3 |
| Attachment | - Experiences in Close Relationship Scale – short (ECR; Wei et al., 2007) | 12 |
| Sleep quality | - Pittsburg Sleep Quality Inventory (PSQI; Buysse et al., 1989) | 18 |
| Covid-19 well-being |  | 4 |
| Covid-19 grief |  | 4 |
| Insufficient integration of the loss | - Experienced Unrealness Scale (EUS; Boelen, 2009) | 5 |
| Negative grief cognitions | - Grief Cognitions Questionnaire (GCQ) | 18 |
| Depressive and anxious avoidance behaviors | - Depressive and Anxious Avoidance in Prolonged Grief Questionnaire (DAAPGD) | 9 |

## 3.3 Full length questionnaires

Below, all questionnaires are presented in their full length, as they were administered to participants in the TABstudy.

Text highlighted in yellow marks items that participants with partner loss have received.

Text highlighted in green marks items that participants with parental loss have received.

### 3.3.1 Background information

**The following section contains a series of questions related to who you are and how you experienced your partner’s/parent’s death.**

1. **What is your age?**

- Write number ________________

2. **What is your gender?**

- Male
- Female

3. **What is the highest level of education you have completed?**

- Primary school, 7th grade
- Primary school, 9th grade
- Primary school, 10th grade
- Upper secondary education (high school)
- Vocational training
- Short-cycle higher education (Academy Profession degree)
- Medium-cycle higher education (Professional bachelor)
- Long-cycle higher education (university degree)

4. **What is your current source of income?**

- Salary
- Pension/early retirement
- Student grant (SU)
- Unemployment benefits
- Sick leave benefits
- Social assistance
- Other

5. **How would you describe your relationship to the person you lost? The deceased was my...**

- Wife
- Husband
- Life partner
- Girlfriend/boyfriend
- Partner
- Cohabitant
- Mother
- Father
- Foster mother
- Foster father
- Stepmother
- Stepfather
- Father’s wife
- Mother’s husband
- Substitute mother
- Substitute father
- Bonus mother
- Bonus father

6. **What was your marital status with your partner?**

- Married
- Cohabiting
- Registered partnership
- Single

→ **If married, cohabiting, in a relationship, or in a registered partnership:**

6a) How many years have you been together with your partner? __________

7. How many years were you together with your partner? __________

8. **Do you have children?**
   *If no, skip to question 9*

- Yes
- No

8a) How many daughters do you have? _____________

8b) How many sons do you have? _____________

9. **How many weeks have passed since your partner’s/parents death?** __________

10. **Was there a prolonged illness leading up to the death?**
    *If no, skip to question 11*

- Yes
- No

10a) How long did this period last (write number of weeks, months, years)? __________

10b) To what extent did you help with caregiving during the illness?

- Not at all
- A little
- To some extent
- Quite a lot
- To an overwhelming extent

10c) How stressful did you find the illness period?

- 1, Not at all (1)
- 2, (2)
- 3, (3)
- 4, Somewhat (4)
- 5, (5)
- 6, (6)
- 7, Extremely (7)

10d) During the illness period, did you feel anxious about becoming seriously ill yourself?

- 1, Not at all (1)
- 2, (2)
- 3, (3)
- 4, Somewhat (4)
- 5, (5)
- 6, (6)
- 7, Extremely (7)

10e) During the illness period, did you feel completely helpless and powerless?

- 1, Not at all (1)
- 2, (2)
- 3, (3)
- 4, Somewhat (4)
- 5, (5)
- 6, (6)
- 7, Extremely (7)

10f) **Where did the illness period mainly take place?**

- Hospital
- At home
- Nursing home
- Hospice
- Other place

10g) **Where did the death occur?**

- Hospital
- At home
- Nursing home
- Hospice
- Other place

11. **What was the cause of your spouse’s/parents death?**

- Old age
- Cancer
- Cardiovascular disease
- Dementia
- Accident
- Suicide
- Diabetes
- Other

### 3.3.2 Single items

#### 3.3.2.1 Single Items at T1

**The following questions are about your daily life after the death of your partner/parent.**

1. **Since the death, have you received help from any of the following?** **Please indicate who you have received help from. You may select more than one answer.**

- Friend
- Female friend
- Boyfriend/girlfriend/spouse/partner
- Parent
- Sister
- Brother
- Daughter
- Son
- Neighbor
- Colleague
- Hospital
- Psychologist
- General practitioner (GP)
- Priest
- Municipality
- Others not listed
- None

(Please indicate multiple answers if applicable)

2. **Have you consumed alcohol during the past week (e.g. a glass of wine or a beer)?**
   *If no, skip to question 3*

- Yes
- No

2a. **What is your usual weekly alcohol consumption?**

- 0-1 drinks
- 2-4 drinks
- 5-7 drinks
- More than 7 drinks
- Not at all

2b. **Since the death, have you experienced an increase in your alcohol consumption?**

- Yes, somewhat
- Yes, very much
- Yes
- No

3. **Have you taken any prescription medication during the past month?**
   *If no, skip to question 4*

- Yes
- No

3a. **What medication have you taken?** _____________________________

3b. **Since the death, have you experienced an increase in your use of medication?**

- Not at all
- Yes, somewhat
- Yes, very much

4. **Do you smoke on a daily basis (e.g. cigarettes, cigars, pipe)?**
   *If no, skip to question 5*.

- Yes
- No

4a. **Since the death, have you experienced an increase in your tobacco consumption?**

- Not at all
- Yes, somewhat
- Yes, very much

5. **Have you lost any other close relatives besides your spouse/parent within the past five years?**
   *If no, go to the next page*

- Yes
- No

5a. **How many other close relatives have you lost besides your spouse/parent?** (Please indicate how many) ________________

#### 3.3.2.2 Single Items at T2

**EVERYDAY LIFE**

**The following questions are about your daily life after the death of your partner/parent.**

1. **Since the death, have you received help from any of the following?** **Please indicate who you have received help from. You may select more than one answer.**

- Friend
- Female friend
- Boyfriend/girlfriend/spouse/partner
- Parent
- Sister
- Brother
- Daughter
- Son
- Neighbor
- Colleague
- Hospital
- Psychologist
- General practitioner (GP)
- Priest
- Municipality
- Others not listed
- None

(Please indicate multiple answers if applicable)

2. **Have you lost any other close relatives besides your spouse/parent within the past few months?**

- Yes
- No

#### 3.3.2.3 Single Items at T3

EVERYDAY LIFE

The following questions are about your daily life after the death of your partner/parent.

1. **Since the death, have you received help from any of the following? Please indicate who you have received help from. You may select more than one answer.**

- Friend
- Female friend
- Boyfriend/girlfriend/spouse/partner
- Parent
- Sister
- Brother
- Daughter
- Son
- Neighbour
- Colleague
- Hospital
- Psychologist
- General practitioner (GP)
- Priest
- Municipality
- Others not listed
- None

(Please indicate multiple answers if applicable)

2. **Have you lost any other close relatives besides your spouse/parent within the past few months?**

- Yes
- No

3. **In the TABstudy, we are also investigating whether psychological treatment can reduce persistent symptoms of grief. If it turns out that you are experiencing persistent grief symptoms when completing this questionnaire, we would like to contact you to tell you more about our offer of free psychological treatment. Only after we have provided more information about the treatment will you decide whether you would like to accept the offer or not. Please indicate whether we may contact you regarding our psychological treatment study.**

- Yes, I would like to hear more.

### 3.3.3 Prolonged Grief (PG-13)

| THOUGHTS AND FEELINGS DURING THE PAST MONTH  Place a check mark indicating your answer next to each question. | | | | | |
| --- | --- | --- | --- | --- | --- |
|  | Not at all | At least once | At least once a week | At least once a day | Several times a day |
| 1. In the past month, how often have you felt yourself longing or yearning for the person you lost? |  |  |  |  |  |
| 2. In the past month, how often have you had intense feelings of emotional pain, sorrow, or pangs of grief related to the lost relationship? |  |  |  |  |  |
|  | | | | Yes | No |
| 3. For questions 1 og 2 above, hav you experienced either if these symptoms at least daily and after 6 months have elapsed since the loss? |  |  |  |  |  |
| 4. In the past month, how often have you tried to avoid reminders that the person you lost is gone? |  |  |  |  |  |
| 5. In the past month, how often have you felt stunned, shocked, or dazed by your loss? |  |  |  |  |  |
|  | Not at all | Slightly | Somewhat | Quite a bit | Overwhelmingly |
| 6. Do you feel confused about your role in life or feel like you don’t know who you are (i.e., feeling that a part of yourself has died)? |  |  |  |  |  |
| 7. Have you had trouble accepting the loss? |  |  |  |  |  |
| 8. Has it been hard for you to trust others since your loss? |  |  |  |  |  |
| 9. Do you feel bitter over your loss? |  |  |  |  |  |
| 10. Do you feel that moving on (e.g., making new friends, pursuing new interests) would be difficult for you now? |  |  |  |  |  |
| 11. Do you feel emotionally numb since your loss? |  |  |  |  |  |
| 12. Do you feel that life is unfulfilling, empty, or meaningless since your loss? |  |  |  |  |  |
|  | | | | Yes | No |
| 13. Have you experienced a significant reduction in social, occupational, or other important areas of functioning (e.g., domestic responsibilities)? |  |  |  |  |  |

### 3.3.4 Prolonged Grief Revised (PG-13-R)

| THOUGHTS AND FEELINGS RIGHT NOW  For each statement below, please indicate how you are feeling right now, either since the death or as a result of the death. | | | | | |
| --- | --- | --- | --- | --- | --- |
| Since the death, or as a result of the death | Not at all | Slightly | Somewhat | Quite a bit | Overwhelmingly |
| 1. Do you feel yourself longing or yearning for the person who died? |  |  |  |  |  |
| 2. Do you have trouble doing the things you normally do because you are thinking so much about the person who died? |  |  |  |  |  |
| 3. Do you feel confused about your role in life or feel like you don´t know who you are any more (i.e., feeling like that at part of you has died)? |  |  |  |  |  |
| 4. Do you have trouble believing that the person who died I really gone? |  |  |  |  |  |
| 5. Do you avoid reminders that the person who died is really gone? |  |  |  |  |  |
| 6. Do you feel emotional pain (e.g.- anger bitterness, sorrow) related to the death? |  |  |  |  |  |
| 7. Do you feel that you have trouble re-engaging in life (e.g., problems engaging with friends, pursuing interests, planning for the future)? |  |  |  |  |  |
| 8. Do you feel emotionally numb or detached from others? |  |  |  |  |  |
| 9. Do you feel that life is meaningless without the person who died? |  |  |  |  |  |
| 10. Do you feel alone or lonely without the deceased? |  |  |  |  |  |
|  | | | | Yes | No |
| 11. Have the symptoms above caused significant impairment in social, occupational, or other important areas of functioning? | | | |  |  |

### 3.3.5 Inventory of Complicated Grief Revised (ICG-R)

*Note*: Selected items included to cover all diagnostic criteria formulated in ICD-11.

| THOUGHTS AND FEELINGS DURING THE PAST MONTH  Please mark the box that best describes how you have been feeling during the past month. | | | | | |
| --- | --- | --- | --- | --- | --- |
|  | Never (0) | Rarely (1) | Sometimes (2) | Often (3) | Always (4) |
| I feel myself longing for the person who died |  |  |  |  |  |
| I feel drawn to places  and things associated  with the person who  died |  |  |  |  |  |
|  | No loneliness | A little loneliness | Some loneliness | Marked loneliness | Overwhelming loneliness |
| I feel lonely a great deal  of the time ever since  s/he died |  |  |  |  |  |
|  | No experience of meaninglessness | A slight experience | Some experience | A marked experience | An overwhelming experience |
| I feel the future is meaningless and without purpose without my spouse/parent. |  |  |  |  |  |
|  | Almost never (less than once a month) | Rarely (monthly) | Sometimes (weekly) | Often (daily) | Always (several times a day) |
| I cannot believe that he/she is dead. |  |  |  |  |  |
|  | No difficulty imagining a satisfying life | A little difficulty | Some difficulty | Marked difficulty | Overwhelming difficulty |
| It is hard for me to imagine a satisfying life without him/her. |  |  |  |  |  |
|  | No change in worldview | Slight change | Some change | Marked change | Overwhelming change |
| I feel that the death has changed my view of the world. |  |  |  |  |  |
|  | Almost never (less than once a month) | Rarely (monthly) | Sometimes (weekly) | Often (daily) | Always (several times a day) |
| I have pain in the same area of my body or I  have some of the same symptoms as the person who died. |  |  |  |  |  |

### 3.3.6 Aarhus Prolonged Grief Disorder Survey (A-PGDs)

| THOUGHTS AND FEELINGS  Please imagine that the name of the person you lost is written on the blank lines (_______) in the questions below, and answer the questions in relation to your loss. | | | | | |
| --- | --- | --- | --- | --- | --- |
|  | **Not at all**  **(1)** | **A little**  **(2)** | **To some extent**  **(3)** | **Very much**  **(4)** | **Overwhel mingly**  **(5)** |
| 1. Have you longed for during the past month? |  |  |  |  |  |
| 2. Have you during the past month found yourself preoccupied with thoughts of even when you did not want to be thinking about them? |  |  |  |  |  |
| 3. Have you had feelings of sadness or sorrow during the past month? |  |  |  |  |  |
| 4. Have you felt guilty during the past month? |  |  |  |  |  |
| 5. Have you felt angry during the past month? |  |  |  |  |  |
| 6. During the past month, has it been hard for you to believe that  is dead? |  |  |  |  |  |
| 7. Have you blamed yourself for your loss during the past month? |  |  |  |  |  |
| 8. During the past month, have you had trouble accepting that  is dead? |  |  |  |  |  |
| 9. During the past month, have you felt that you have lost a part of yourself?  *(e.g.* feeling as though a part of you has died*)* |  |  |  |  |  |
| 10. During the past month, have you been unable to experience positive emotions? |  |  |  |  |  |
| 11. During the past month, have you felt emotionally numb?  *(e.g., having difficulties with feeling emotions as you used to do, being emotionally stunned)* |  |  |  |  |  |
| 12. Have you had difficulty engaging in social or other activities during the past month? |  |  |  |  |  |
| 13. Have you felt loneliness during the past month? |  |  |  |  |  |
| 14. During the past month, have you tried to avoid reminders that  is dead? *(e.g., avoiding certain thoughts, feelings, places, music, conversation topics, etc. or keeping yourself constantly going)* |  |  |  |  |  |
| 15. During the past month, have you felt that life is meaningless since  has died? |  |  |  |  |  |
| 16. Have you felt bitterness during the past month? |  |  |  |  |  |
| **17. Overall, have these difficulties led to a decline in your level of functioning?**  ***(i.e., your ability to function in everyday life)*** |  |  |  |  |  |
|  | | | |  |  |
| ***If you answered "A little / (2)" or higher to question 17:*** | | | | **YES** | **NO** |
| 17a. Does this apply in relation to your work/study/daily tasks? | | | |  |  |
| 17b. Does this apply to your social life? | | | |  |  |
| 17c. Does this apply to your family life/domestic obligations? | | | |  |  |
| 17d. Does this apply to other areas than those mentioned? | | | |  |  |
|  | | | |  |  |
|  | | | | **YES** | **NO** |
| 18. Have any of your acquaintances expressed concern about your grief reaction?  *(e.g., that they feel that it exceeds what they consider normal in relation to your social, cultural or religious norms)* | | | |  |  |
| 19. Are you worried about your own grief reaction, including that it is more severe or intense than you expected?  *(e.g., compared to the people you surround yourself with or what you think is normal)* | | | |  |  |
| 20. Would you say that you felt this way during the past 6 months? (Please answer the question in relation to your total response to the questions above) | | | |  |  |

### 3.3.7 Center for Epidemiologic Studies Short Depression Scale (CESD-10)

| FEELINGS AND ACTIONS DURING THE PAST WEEK  Below is a list of ways you may have felt or behaved. Please indicate how often you have felt this way during the past week by checking the corresponding response. | | | | |
| --- | --- | --- | --- | --- |
|  | **Rarely or none of the time (less than 1 day)** | **Some or a little of the time**  **(1-2 days)** | **Occasionally or a moderate amount of time (3-4 days)** | **All of the time**  **(5-7 days)** |
| 1. I was bothered by things that usually don't bother me. |  |  |  |  |
| 2. I had trouble keeping my mind on what I was doing. |  |  |  |  |
| 3. I felt depressed |  |  |  |  |
| 4. I felt that everything I did was an effort. |  |  |  |  |
| 5. I felt hopeful about the future. |  |  |  |  |
| 6. I felt fearful. |  |  |  |  |
| 7. My sleep was restless. |  |  |  |  |
| 8. I was happy. |  |  |  |  |
| 9. I felt lonely. |  |  |  |  |
| 10. I could not “get going” |  |  |  |  |

### 3.3.8 Generalized Anxiety Disorder (GAD-7)

| MOOD DURING THE PAST 14 DAYS  How often during the past 14 days have you been bothered by the following problems? | | | | |
| --- | --- | --- | --- | --- |
|  | Not at all  (0) | Several days  (1) | More than half of the days  (2) | Nearly every day (3) |
| 1. Feeling nervous, anxious, or on edge |  |  |  |  |
| 2. Not being able to stop or control worrying |  |  |  |  |
| 3. Worrying too much about different things |  |  |  |  |
| 4. Trouble relaxing |  |  |  |  |
| 5. Being so restless that it is hard to sit still |  |  |  |  |
| 6. Becoming easily annoyed or irritable |  |  |  |  |
| 7. Feeling afraid, as if something awful might happen |  |  |  |  |

### 3.3.9 PTSD Check List – Civilian version (PCL-5)

| THOUGHTS DURING THE PAST MONTH  Below is a list of problems that people sometimes experience after the death of someone they care about. Please read each problem carefully and indicate how much it has bothered you during the past month. | | | | | |
| --- | --- | --- | --- | --- | --- |
|  | Not at all (0) | A little bit (1) | Moderately (2) | Quite a bit (3) | Extremely (4) |
| 1. Repeated, disturbing, and unwanted memories of the stressful experience? |  |  |  |  |  |
| 2. Repeated, disturbing dreams of the stressful experience? |  |  |  |  |  |
| 3. Suddenly feeling or acting as if the stressful experience were actually happening again (as if you were actually back there reliving it)? |  |  |  |  |  |
| 4. Feeling very upset when something reminded you of the stressful experience? |  |  |  |  |  |
| 5. Having strong physical reactions when something reminded you of the stressful experience (for example, heart pounding, trouble breathing, sweating)? |  |  |  |  |  |
| 6. Avoiding memories, thoughts, or feelings related to the stressful experience? |  |  |  |  |  |
| 7. Avoiding external reminders of the stressful experience (for example, people, places, conversations, activities, objects, or situations)? |  |  |  |  |  |
| 8. Trouble remembering important parts of the stressful experience? |  |  |  |  |  |
| 9. Having strong negative beliefs about yourself, other people, or the world (for example, having thoughts such as: I am bad, there is something seriously wrong with me, no one can be trusted, the world is completely dangerous)? |  |  |  |  |  |
| 10. Blaming yourself or someone else for the stressful experience or what happened after it? |  |  |  |  |  |
| 11. Having strong negative feelings such as fear, horror, anger, guilt, or shame? |  |  |  |  |  |
| 12. Loss of interest in activities that you used to enjoy? |  |  |  |  |  |
| 13. Feeling distant or cut off from other people? |  |  |  |  |  |
| 14. Trouble experiencing positive feelings (for example, being unable to feel happiness or have loving feelings for people close to you)? |  |  |  |  |  |
| 15. Irritable behavior, angry outbursts, or acting aggressively? |  |  |  |  |  |
| 16. Taking too many risks or doing things that could cause you harm? |  |  |  |  |  |
| 17. Being “super alert” or watchful or on guard? |  |  |  |  |  |
| 18. Feeling jumpy or easily startled? |  |  |  |  |  |
| 19. Having difficulty concentrating? |  |  |  |  |  |
| 20. Trouble falling or staying asleep? |  |  |  |  |  |

### 3.3.10 Well-being Scale (WHO-5)

| WELL-BEING DURING THE PAST 2 WEEKS  Please indicate, for each of the 5 statements, the box that best describes how you have been feeling during the past two weeks.  Note that a higher number indicates better well-being.  In the past two weeks... | | | | | | |
| --- | --- | --- | --- | --- | --- | --- |
|  | All of the time | Most of the time | More than half of the time | Less than half of the time | Some of the time | At no time |
| I have felt cheerful and in good spirits |  |  |  |  |  |  |
| I have felt calm and relaxed |  |  |  |  |  |  |
| I have felt active and vigorous |  |  |  |  |  |  |
| I woke up feeling fresh and rested |  |  |  |  |  |  |
| My daily life has been full of things that interested me |  |  |  |  |  |  |

### 3.3.11 Three Item Loneliness Scales (T-ILS)

| SOCIAL CONNECTIONS  The following questions concern how you feel about different aspects of your life.  For each question, please indicate how often you feel this way. | | | |
| --- | --- | --- | --- |
|  | Hardly ever | Some of the time | Often |
| How often do you feel isolated from others? |  |  |  |
| How often do you feel left out? |  |  |  |
| How often do you feel that you lack companionship? |  |  |  |

### 3.3.12 Crisis Social Support (CSS)

| SOCIAL NETWORK  How do you currently experience your surroundings? | | | | | | | |
| --- | --- | --- | --- | --- | --- | --- | --- |
|  | Never (1) | (2) | (3) | Sometimes (4) | (5) | (6) | Always (7) |
| When you need to talk, how often is someone willing to listen? |  |  |  |  |  |  |  |
| Do you have contact with others in the same situation or with similar experiences? |  |  |  |  |  |  |  |
| Are you able to talk about your thoughts and feelings related to the death? |  |  |  |  |  |  |  |
| Do people show sympathy and support? |  |  |  |  |  |  |  |
| Is there someone who helps you with practical matters? |  |  |  |  |  |  |  |
| Do you sometimes feel let down by people you expected would support you? |  |  |  |  |  |  |  |
| Overall, are you satisfied with the support you receive? |  |  |  |  |  |  |  |

### 3.3.13 Centrality of Event Scale (CES)

| EXPERIENCE OF LOSS  Please think back to your spouse’s/parent’s death and answer the following questions as honestly as you can by marking a number between 1 and 5. | | | | | |
| --- | --- | --- | --- | --- | --- |
|  | Totally disagree (1) | Disagree (2) | Neutral (3) | Agree (4) | Totally agree (5) |
| I feel that this event has become part of my identity. |  |  |  |  |  |
| This event has become a reference point for the way I understand myself and the world. |  |  |  |  |  |
| I feel that this event has become a central part of my life story. |  |  |  |  |  |
| This event has colored the way I think and feel about other experiences. |  |  |  |  |  |
| This event permanently changed my life. |  |  |  |  |  |
| I often think about the effects this event will have on my future. |  |  |  |  |  |
| This event was a turning point in my life. |  |  |  |  |  |

### 3.3.14 12-Item Short Form Health Survey (SF-12)

ACTIVITY AND HEALTH
The following questions concern your perception of your health. The information will help provide an overview of how you are feeling and how well you are able to carry out your daily activities.
Please answer each question by marking the response that best describes you. If you are unsure how to answer, please respond as best you can.

**1. In general, would you say your health is:**

- Execellent
- Very good
- Good
- Fair
- Poor

The following items are about activities you might do during a typical day. Does your health now limit you in these activities? If so, how much?

**2. Moderate activities such as moving a table, pushing a vacuum cleaner, bowling or playing golf.**

- Yes, limited a lot
- Yes, limited a little
- No, not limited at all

**3. Climbing several flights of stairs**

- Yes, limited a lot
- Yes, limited a little
- No, not limited at all

During the past 4 weeks, have you had any of the following problems with your work or other regular daily activities as a result of your physical health?

1. **Accomplished less than you would like**

- Yes
- No

2. **Were limited in the kind of work or other activities?**

- Yes
- No

During the past 4 weeks, have you had any of the following problems with your work or other regular daily activities as a result of any emotional problems (such as feeling depressed or anxious)?

3. **Accomplished less than you like?**

- Yes
- No

4. **Didn’t do work or other activities as carefully as usual**

- Yes
- No

5. **During the past 4 weeks how much did pain interfere with your normal work (including both work outside the home and housework)**

- Not at all
- A little bit
- Moderately
- Quite a bit
- Extremely

These questions are about how you feel and how things have been with you during the past 4 weeks. For each question, please give the one answer that comes closest to the way you have been feeling. How much of the time during the past 4 weeks……

6. **Have you felt calm and peaceful?**

- All of the time
- Most of the time
- A good bit of the time
- Some of the time
- A little of the time
- None of the time

7. **Did you have a lot of energy**

- All of the time
- Most of the time
- A good bit of the time
- Some of the time
- A little of the time
- None of the time

8. **Have you felt downhearted and blue?**

- All of the time
- Most of the time
- A good bit of the time
- Some of the time
- A little of the time
- None of the time

9. **During the past 4 weeks, how much of the time has you physical health or emotional problems interfered with your social activities (like visiting friends, relatives, etc.)?**

- All of the time
- Most of the time
- A good bit of the time
- Some of the time
- A little of the time
- None of the time

### 3.3.15 Life Changes

**Have you experienced any of the following events since the death of your spouse/parent?**
You may select more than one event from the list below.

- Moved to a new home
- Changed workplace
- Unemployed
- Retired
- Started a new relationship
- Diagnosed with a critical or serious illness
- A close relative diagnosed with a critical or serious illness
- Death of a close family member or friend
- Taken up a new hobby or leisure activity (e.g., choir, football, gymnastics)
- Became a grandparent or great-grandparent
- Became a parent or got pregnant
- Divorce/broke up with boy/girlfriend
- Lost a pet
- Got a new pet
- Other: _______________________________________________

*(Please describe in your own words)*

**To what extent would you say your life has been affected by the events you have experienced?**
(Please only respond to the events that apply to you)

|  | Not at all | A little | Moderately | Quite a lot | Extremely |
| --- | --- | --- | --- | --- | --- |
| Moved to a new home |  |  |  |  |  |
| Changed workplace |  |  |  |  |  |
| Unemployed |  |  |  |  |  |
| Diagnosed with a critical or serious illness |  |  |  |  |  |
| A close relative diagnosed with a critical or serious illness |  |  |  |  |  |
| Death of a close family memer or friend |  |  |  |  |  |
| Taken up a new hobby or leisure activity |  |  |  |  |  |
| Became a grandparents or great-grandparent |  |  |  |  |  |
| Had a child or got pregnant |  |  |  |  |  |
| Divorce/broke up with boy/girlfriend |  |  |  |  |  |
| Lost a pet |  |  |  |  |  |
| Got a new pet |  |  |  |  |  |
| Other (please specify) _____________ |  |  |  |  |  |

### 3.3.16 Rumination-Reflection Questionnaire (RRQ)

| PERSONAL ATTITUDES AND WAYS OF BEING For each of the statements below, please indicate with a check mark how much you agree or disagree. | | | | | |
| --- | --- | --- | --- | --- | --- |
|  | Strongly Disagree | Disagree | Neutral | Agree | Strongly Agree |
| My attention is often focused on aspects of myself I wish I'd stop thinking about |  |  |  |  |  |
| I always seem to be "re-hashing" in my mind recent things I've said or done |  |  |  |  |  |
| Sometimes it is hard for me to shut off thoughts about myself |  |  |  |  |  |
| Long after an argument or disagreement is over with, my thoughts keep going back to what happened |  |  |  |  |  |
| I tend to "ruminate" or dwell over things that happen to me for a really long time afterward |  |  |  |  |  |
| I don't waste time re-thinking things that are over and done with |  |  |  |  |  |
| Often I'm playing back over in my mind how I acted in a past situation |  |  |  |  |  |
| I often find myself re-evaluating something I've done |  |  |  |  |  |
| I never ruminate or dwell on myself for very long |  |  |  |  |  |
| It is easy for me to put unwanted thoughts out of my mind |  |  |  |  |  |
| I often reflect on episodes in my life that I should no longer concern myself with |  |  |  |  |  |
| I spend a great deal of time thinking back over my embarrassing or disappointing moments. |  |  |  |  |  |

### 3.3.17 Experience in Close Relationship Scale - short (ECR)

| YOUR RELATIONSHIP WITH YOUR PARTNER The following statements concern how you experienced your romantic relationship. We are interested in how you perceived your relationship with the partner you have lost. Please answer the questions as best you can based on how you felt in that relationship. For each statement, indicate the degree to which you agree or disagree. | | | | | | | |
| --- | --- | --- | --- | --- | --- | --- | --- |
| YOUR RELATIONSHIP WITH OTHERS The following statements concern how you experience being in a romantic relationship. We are interested in how you generally perceive relationships — not just your current or most recent one. Please answer the questions as accurately as you can based on how things usually are for you when you are in a relationship. For each statement, indicate the degree to which you agree or disagree. | | | | | | | |
|  | Strongly disagree | Disagree | Somewhat disagree | Neither agree nor disagree | Somewhat agree | Agree | Strongly agree |
| It helped/helps to turn to my romantic partner in times of need |  |  |  |  |  |  |  |
| I needed/need a lot of reassurance that my partner loved me |  |  |  |  |  |  |  |
| I wanted/want to get close to my partner, but I kept pulling back |  |  |  |  |  |  |  |
| I found/find that my partner didn’t/don’t want to get as close as I would like |  |  |  |  |  |  |  |
| I turned/turn to my partner for many things, including comfort and reassurance |  |  |  |  |  |  |  |
| My desire to be very close sometimes scared/scare my partner away |  |  |  |  |  |  |  |
| I tried/try to avoid getting too close to my partner |  |  |  |  |  |  |  |
| I didn’t/don’t often worry about being abandoned |  |  |  |  |  |  |  |
| I usually discussed/discuss my problems and concerns with my partner |  |  |  |  |  |  |  |
| I got/get frustrated if my romantic partner was/is not available when I needed/need him/her |  |  |  |  |  |  |  |
| I was/become nervous when my partner got/get too close to me |  |  |  |  |  |  |  |
| I worried/worry that my romantic partner wouldn’t/will not care about me as much as I cared/care about him/her |  |  |  |  |  |  |  |

### 3.3.18 Life Orientation Test – Revised (LOT-R)

| PERSONAL ATTITUDES AND WAYS OF BEING Please indicate how much you agree with the following 10 statements. | | | | | |
| --- | --- | --- | --- | --- | --- |
|  | Strongly disagree | Disagree | Neutral | Agree | Strongly agree |
| In uncertain times, I usually expect the best |  |  |  |  |  |
| It’s easy for me to relax. |  |  |  |  |  |
| If something can go wrong for me, it will |  |  |  |  |  |
| I’m always optimistic about my future |  |  |  |  |  |
| I enjoy my friends a lot |  |  |  |  |  |
| It’s important for me to keep busy |  |  |  |  |  |
| I hardly ever expect things to go my way |  |  |  |  |  |
| I don’t get upset too easily |  |  |  |  |  |
| I rarely count on good things happening to me |  |  |  |  |  |
| Overall, I expect more good things to happen to me than bad |  |  |  |  |  |

### 3.3.19 NEO Personality Inventory – Revised: Neuroticism only (NEO-PI-R)

| PERSONAL ATTITUDES AND WAYS OF BEING Would you please read the following statements and indicate how well each one describes you? | | | | | |
| --- | --- | --- | --- | --- | --- |
|  | Strongly disagree | Disagree | Neutral | Agree | Strongly agree |
| I am a well-balanced person |  |  |  |  |  |
| Sometimes I feel like I’m not good at anything |  |  |  |  |  |
| I rarely feel anxious or uneasy |  |  |  |  |  |
| I am rarely depressed or sad |  |  |  |  |  |
| At times, I’ve felt so ashamed that I just wanted to hide |  |  |  |  |  |
| When under a lot of pressure, I sometimes feel like I’m falling apart |  |  |  |  |  |
| I often feel tense and nervous |  |  |  |  |  |
| Compared to others, I often feel inferior |  |  |  |  |  |
| I often find it difficult to make decisions |  |  |  |  |  |
| Sometimes I do things impulsively that I later regret |  |  |  |  |  |
| Even small irritations can make me feel frustrated |  |  |  |  |  |
| I am always able to control my emotions |  |  |  |  |  |

### 3.3.20 Flexible Regulation of Emotional Expression (FREE)

POSITIVE AND NEGATIVE EMOTIONS
Expressing emotions is a natural part of everyday life. For social reasons, we may sometimes need to express what we feel more strongly than we actually do, and at other times we may need to tone down or hide what we truly feel.

The following scenarios involve POSITIVE emotion. For each scenario, indicate how well you would be able to be even MORE EXPRESSIVE than usual of how you were feeling:

|  | Unable (1) | 2 | 3 | 4 | 5 | Very able (6) |
| --- | --- | --- | --- | --- | --- | --- |
| A coworker gets a promotion and wants to talk about it. |  |  |  |  |  |  |
| You receive a gift from a family member but it’s a shirt you dislike. |  |  |  |  |  |  |

The following scenarios involve NEGATIVE emotion. For each scenario, indicate how well you would be able to be even MORE EXPRESSIVE than usual of how you were feeling:

|  | Unable (1) | 2 | 3 | 4 | 5 | Very able (6) |
| --- | --- | --- | --- | --- | --- | --- |
| Your friend is telling you about what a terrible day they had. |  |  |  |  |  |  |
| You’re attending the funeral of someone you don’t know. |  |  |  |  |  |  |

The following scenarios involve POSITIVE emotion. For each scenario, indicate how well you would be able to CONCEAL how you were feeling:

|  | Unable (1) | 2 | 3 | 4 | 5 | Very able (6) |
| --- | --- | --- | --- | --- | --- | --- |
| While having dinner with a friend who has just recently lost their job, you receive a phone call from your boss stating you will get a raise. |  |  |  |  |  |  |
| You’re a guest at a solemn religious ceremony and the person sitting next to you just whispered a funny joke. |  |  |  |  |  |  |

The following scenarios involve NEGATIVE emotion. For each scenario, indicate how well you would be able to CONCEAL how you were feeling:

|  | Unable (1) | 2 | 3 | 4 | 5 | Very able (6) |
| --- | --- | --- | --- | --- | --- | --- |
| You are at a social event and the person you’re talking to frequently spits while they speak. |  |  |  |  |  |  |
| You have just heard about the death of a close relative right before an important work meeting. |  |  |  |  |  |  |

### 3.3.21 Emotion Regulation Questionnaire (ERQ)

| MANAGING EMOTIONS We would like to ask you some questions about your emotional life — specifically, how you control (that is, regulate and manage) your emotions. The questions below relate to two different aspects of your emotional life. One concerns your emotional experience — what you feel on the inside. The other concerns your emotional expression — how you show your feelings through the way you speak, gesture, and behave. Although some of the following questions may appear similar, they address important differences. For each question, please indicate your response. | | | | | | | |
| --- | --- | --- | --- | --- | --- | --- | --- |
|  | Strongly disagree (1) | (2) | (3) | Neutral (4) | (5) | (6) | Strongly agree (7) |
| When I want to feel more *positive* emotion (such as joy or amusement), I *change*  *what I’m thinking about*. |  |  |  |  |  |  |  |
| I keep my emotions to myself |  |  |  |  |  |  |  |
| When I want to feel less *negative* emotion (such as sadness or anger), I *change*  *what I’m thinking about*. |  |  |  |  |  |  |  |
| When I am feeling *positive* emotions, I am careful not to express them. |  |  |  |  |  |  |  |
| When I’m faced with a stressful situation, I make myself *think about it* in a way  that helps me stay calm. |  |  |  |  |  |  |  |
| I control my emotions by *not expressing them*. |  |  |  |  |  |  |  |
| When I want to feel more *positive* emotion, I *change the way I’m thinking* about  the situation. |  |  |  |  |  |  |  |
| I control my emotions by *changing the way I think* about the situation I’m in. |  |  |  |  |  |  |  |
| When I am feeling *negative* emotions, I make sure not to express them. |  |  |  |  |  |  |  |
| When I want to feel less *negative* emotion, I *change the way I’m thinking* about  the situation. |  |  |  |  |  |  |  |

### 3.3.22 Transactive Memory System (TMS)

| COOPERATION IN DAILY LIFE We would now like you to think back to how you and your partner/parent worked together in daily life when something needed to be done. Please consider any kind of everyday tasks where you had to cooperate to get things done — for example, planning a trip or outing, going shopping together, assembling furniture, or doing household chores. | | | | | |
| --- | --- | --- | --- | --- | --- |
|  | Strongly disagree | Disagree | Neither agree nor disagree | Agree | Strongly agree |
| My partner/parent and I had specialized knowledge in specific areas. |  |  |  |  |  |
| I had knowledge in a specific area that my partner/parent did not have. |  |  |  |  |  |
| My partner/parent and I were each responsible for having expertise in different areas. |  |  |  |  |  |
| The specialized knowledge that both my partner/parent and I had was necessary to complete tasks. |  |  |  |  |  |
| **I knew whether it was my partner/parent or myself who had the relevant expertise in a given area.** |  |  |  |  |  |
| **I felt comfortable accepting suggestions from my partner/parent.** |  |  |  |  |  |
| **I trusted that my partner’s/parents knowledge was reliable.** |  |  |  |  |  |
| **I felt comfortable relying on the information my partner/parent gave me.** |  |  |  |  |  |
| **When my partner/parent gave me information, I felt the need to double-check it myself.** |  |  |  |  |  |
| **I had a high level of confidence in my partner’s/parent’s expertise.** |  |  |  |  |  |
| **My partner/parent and I worked together in a well-coordinated manner** |  |  |  |  |  |
| **My partner/parent and I had very few misunderstandings when completing tasks.** |  |  |  |  |  |
| **My partner/parent and I usually completed tasks successfully on the first try.** |  |  |  |  |  |
| **My partner/parent and I were able to complete tasks smoothly and efficiently** |  |  |  |  |  |
| **There was little confusion when my partner/parent and I began working on a task together.** |  |  |  |  |  |

### 3.3.23 Everyday Memory Questionnaire (EMQ)

| DAILY ACTIVITIES Below is a list of examples of things that may happen to people in everyday life. Some of them may happen frequently, while others may occur only rarely. We would like to know how often, on average, you think each example has happened to you over the past month. Please choose the response that best reflects your experience. | | | | | |
| --- | --- | --- | --- | --- | --- |
|  | Once or less in the last month. | More than once a month but less than once a week. | About once a week. | More than once a week or less than once a day | Once or more in a day. |
| Having to check whether you have done something that you should have done. |  |  |  |  |  |
| Forgetting when it was that something happened; for example, whether it was  yesterday or last week. |  |  |  |  |  |
| Forgetting that you were told something yesterday or a few days ago, and maybe  having to be reminded about it. |  |  |  |  |  |
| Starting to read something (a book or an article in a newspaper, or a magazine)  without realizing you have already read it before. |  |  |  |  |  |
| Finding that a word is ‘on the tip of your tongue’. You know what it is but cannot quite find it. |  |  |  |  |  |
| Completely forgetting to do things you said you would do, and things you planned  to do. |  |  |  |  |  |
| Forgetting important details of what you did or what happened to you the day before. |  |  |  |  |  |
| When talking to someone, forgetting what you have just said. Maybe saying  ‘what was I talking about?’ |  |  |  |  |  |
| When reading a newspaper or magazine, being unable to follow the thread of a story; losing track of what it is about. |  |  |  |  |  |
| Forgetting to tell somebody something important, perhaps forgetting to pass on a  message or remind someone of something. |  |  |  |  |  |
| Getting the details of what someone was told you mixed up and confused. |  |  |  |  |  |
| Forgetting where things are normally kept or looking for them in the wrong place. |  |  |  |  |  |
| Repeating to someone what you have just told them or asking someone the same question twice. |  |  |  |  |  |

### 3.3.24 Scale for Inclusion of Others in Self (IOS)

RELATIONSHIPS WITH OTHERS
One way to describe the relationships we have with other people is to consider how much they overlap with our sense of self — that is, how much you feel that the other person is part of who you are.

**When you think about your partner/parent before he/she died, how much did your sense of self overlap with him/her?**
Please circle the image that best describes your past relationship with your partner/parent.

**And when you think about your partner/parent now, after his/her death, how much do you still feel that your sense of self overlaps with him/her?**
Please circle the image that best describes your current relationship with your partner/parent.

### 3.3.25 Loss Chapter

CHAPTERS OF LIFE
In this part of the study, we ask you to describe the period or chapter of your life that relates to the loss of your partner/parent.

Please include descriptions of:

- your relationship with your partner/parent
- what happened both in the time leading up to the loss and in the months that followed,
- what you thought and felt during this time,
- what this period says about you as a person, and
- how this period may have changed you.

Now, once again, please think about the period or chapter in your life when you lost your partner/parent.

|  | Not at all | To a low degree | To some degree | To at high degree | To a very high degree |
| --- | --- | --- | --- | --- | --- |
| Think about how you experienced the period at the time it happened. To what extent would you describe what took place during that time as something positive? |  |  |  |  |  |
| Think about how you experienced the period at the time it happened. To what extent would you describe what took place during that time as something negative? |  |  |  |  |  |
| Think about how you experience the period today. Does it highlight any positive qualities that characterize you today? |  |  |  |  |  |
| Think about how you experience the period today. Does it highlight any negative qualities that characterize you today? |  |  |  |  |  |
| Think about how you experience the period today. Has the period led to personal change in a positive way? |  |  |  |  |  |
| Think about how you experience the period today. Has the period led to personal change in a negative way? |  |  |  |  |  |

### 3.3.26 Existential Feeling in Grief (EFS)

There is currently no validated English translation of this questionnaire.

### 3.3.27 Bereavement Hallucinations

SENSORY EXPERIENCES

It often happens that people who have lost a close loved one spontaneously experience sensory perceptions of the deceased. They may see, hear, feel, smell, taste, or sense his or her presence.

Note: This does not refer to vivid dreams or experiences through a clairvoyant or medium.

Please answer the questions as best you can. There are no right or wrong answers.

1. Since the loss, have you experienced sensing your deceased spouse through one of the five senses or by feeling his/her presence while awake?

- Yes
- No

2. In what way(s) have you sensed your deceased spouse while awake since the loss?

- Seen him/her
- Heard his/her voice
- Heard sounds (e.g., footsteps)
- Felt physical contact (e.g., kiss/hug)
- Sensed his/her presence (e.g., as a general presence or a feeling of being nearby)
- Taste experience (e.g., tasted a familiar dish without food being present)
- Smelled him/her (e.g., their scent without knowing where it came from, such as from clothing)

3. How often have you sensed your deceased spouse while awake during the past year?

The experiences have stopped and have not occurred in the past year

- At least once in the past year
- At least twice in the past year
- At least once a month in the past year
- At least once a week in the past year
- At least once a day in the past year
- All the time

### 3.3.28 Experienced Unrealness Scales (EUS)

| EXPERIENCE OF THE LOSS To what extent have the following statements applied to you during the past month? | | | | | | | | |
| --- | --- | --- | --- | --- | --- | --- | --- | --- |
|  | Not at all true for me (1) | (2) | (3) | (4) | (5) | (6) | (7) | Completely true for me (8) |
| I have trouble believing that I will never see ____ again, |  |  |  |  |  |  |  |  |
| It feels unreal that ____ is gone forever |  |  |  |  |  |  |  |  |
| Rationally I know, but emotionally it feels unreal, that _____ is gone forever |  |  |  |  |  |  |  |  |
| I still can hardly imagine that ______ will never be here again |  |  |  |  |  |  |  |  |
| Sometimes it feels as if _______ is just temporarily gone and will return again soon |  |  |  |  |  |  |  |  |

### 3.3.29 Grief Cognitions Questionnaire (GCQ)

| EXPERIENCE OF THE LOSS Below you will find a series of negative beliefs. Please indicate the extent to which you agree with each of these beliefs. | | | | | | |
| --- | --- | --- | --- | --- | --- | --- |
|  | Strongly disagree (0) | (1) | (2) | (3) | (4) | Strongly agree (5) |
| Since he/she is dead, I think I am worthless. |  |  |  |  |  |  |
| Since he/she died, I realize that the world is a bad place. |  |  |  |  |  |  |
| The people around me should give me more support. |  |  |  |  |  |  |
| If I let go of my emotions, I will go crazy |  |  |  |  |  |  |
| His/her death has made me realize that we live in an awful world. |  |  |  |  |  |  |
| My grief reactions are abnormal. |  |  |  |  |  |  |
| I don’t have confidence in the future. |  |  |  |  |  |  |
| As long as I mourn, I maintain the bond with him/her |  |  |  |  |  |  |
| My life is useless since he/she died. |  |  |  |  |  |  |
| I should have prevented his/her death |  |  |  |  |  |  |
| My life is meaningless since he/she died. |  |  |  |  |  |  |
| If I had done things differently, he/she would still be alive. |  |  |  |  |  |  |
| Ever since he/she died, I think negatively about myself. |  |  |  |  |  |  |
| I do not react to this loss normally |  |  |  |  |  |  |
| As long as I mourn, I do not really have to let him/her go. |  |  |  |  |  |  |
| People around me should show much more interest in me. |  |  |  |  |  |  |
| Since he/she is no longer here, I have a negative view on the future. |  |  |  |  |  |  |
| If I allow my feelings to come, I will lose control. |  |  |  |  |  |  |

### 3.3.30 Depressive and Anxious Avoidance in Prolonged Grief Questionnaire (DAAPGD)

| EXPERIENCE OF THE LOSS To what extent have the following statements applied to you during the past month? | | | | | | | | |
| --- | --- | --- | --- | --- | --- | --- | --- | --- |
|  | Not at all true for me  (1) | (2) | (3) | (4) | (5) | (6) | (7) | Completely true for me (8) |
| Since _____ is dead, I do much less of the things that I used to enjoy. |  |  |  |  |  |  |  |  |
| Since ____ died, I avoid activities that used to give me satisfaction, because these activities now seem meaningless to me |  |  |  |  |  |  |  |  |
| I avoid doing activities that used to bring me pleasure, because I feel unable to carry out these activities. |  |  |  |  |  |  |  |  |
| I develop very few new activities since ______ died, because I am unable to do so. |  |  |  |  |  |  |  |  |
| Since ______ died, there are several activities, hobbies, and acquaintances that I pay much less attention to. |  |  |  |  |  |  |  |  |
| I avoid to dwell on the fact that _____ is dead and will never return. |  |  |  |  |  |  |  |  |
| I avoid situations and places that confront me with the fact that _____ is dead and will never return. |  |  |  |  |  |  |  |  |
| I avoid to dwell on painful thoughts and memories connected to his/her death. |  |  |  |  |  |  |  |  |
| I deliberately retrieve positive memories related to ______ as a means to avoid thinking about the fact that ______ is dead and will never return. |  |  |  |  |  |  |  |  |

### 3.3.31 Pittsburg Sleep Quality Inventory (PSQI)

SLEEP DURING THE PAST MONTH
The following questions concern your sleeping habits over the past month. Please indicate the response that most accurately reflects how you have been most days during the past month.
Kindly answer all questions.

1. **During the past month, what time have you usually gone to bed at night?**

- Bed time _________

2. **During the past month, how long (in minutes) has it usually taken you to fall asleep each night?**

- Number of minutes _____________

3. **During the past month, what time have you usually gotten up in the morning?**

- Getting up time ________

4. **During the past month, how many hours of actual sleep did you get at night? (This may be different than the number of hours you spent in bed)**

- Hours of sleep per night _________

5. **For each of the following questions, please check the box that best describes how you have been.**

**During the past month, how often have you had trouble sleeping because you...**

|  | Not during the past month | Less than once a week | Once or twice a week | Three or more times a week |
| --- | --- | --- | --- | --- |
| Cannot sleep within 30 minutes |  |  |  |  |
| Wake up in the middle of the night or early morning |  |  |  |  |
| Have to get up to use the bathroom |  |  |  |  |
| Cannot breathe comfortably |  |  |  |  |
| Cough or snore loudly |  |  |  |  |
| Feel too cold |  |  |  |  |
| Feel too hot |  |  |  |  |
| Had bad dreams |  |  |  |  |
| Have pain |  |  |  |  |
| Other reason(s), please describe ____________________________________ |  |  |  |  |

6. **During the past month, how would you rate your sleep quality overall?**

- Very good
- Fairly good
- Fairly bad
- Very bad

7. **During the past month, how often have you taken medicine to help you sleep (prescribed or “over the counter”)?**

- Not during the past month
- Less than once a week
- Once or twice a week
- Tree or more times a week

8. **During the past month, how often have you had trouble staying awake while driving, eating, meals or engaging in social activity?**

- Not during the past month
- Less than once a week
- Once or twice a week
- Tree or more times a week

9. **During the past month, how much of a problem has it been for you to keep up enough enthusiasm to get things done?**

- No problem at all
- Only a very slight problem
- Somewhat of a problem
- A very big problem

### 3.3.32 Covid-19 Well-being

WELL-BEING DURING THE COVID-19 PANDEMIC

**Has the COVID-19 situation affected your well-being over the past year?**

- Yes
- No

*If yes, please describe how in the space below:*

**To what extent has the COVID-19 situation affected your well-being positively?**

- Not at all (1)
- (2)
- To some extent (3)
- (4)
- To a very high extent (5)

**To what extent has the COVID-19 situation affected your well-being negatively?**

- Not at all (1)
- (2)
- To some extent (3)
- (4)
- To a very high extent (5)

### 3.3.33 Covid-19 Grief

GRIEF DURING THE COVID-19 PANDEMIC

Has the COVID-19 situation affected your grief response over the past year?

- Yes
- No

### 3.3.34 Press Inquiry

PRESS INQUIRIES
There is great interest in the Danish media in the life situations of bereaved individuals and the grief they experience after a loss.
From time to time, we are asked whether participants in the study might be willing to speak to the press.

**May we contact you in connection with inquiries from journalists?**

- **No, I do not wish to be contacted**
- **Yes, you may contact me and tell me more about what the media inquiry involves**

(*Your personal information will not be shared directly with the journalist, and you are under no obligation to participate. We will contact you with more details, and you are of course always free to decline.*)
